# A Brief Molecular History of Vγ9Vδ2 TCR‐Mediated Phosphoantigen Sensing

**DOI:** 10.1111/imr.70023

**Published:** 2025-04-03

**Authors:** Fiyaz Mohammed, Carrie R. Willcox, Benjamin E. Willcox

**Affiliations:** ^1^ Department of Immunology and Immunotherapy, School of Infection, Inflammation and Immunology, College of Medicine and Health University of Birmingham Birmingham UK; ^2^ Cancer Immunology and Immunotherapy Centre, College of Medicine and Health University of Birmingham Birmingham UK; ^3^ National Institute for Health and Care Research (NIHR) Birmingham Biomedical Research Centre Birmingham UK

**Keywords:** butyrophilin, innate‐like, phosphoantigen, T cell receptor, Vγ9Vδ2 T cells

## Abstract

Vγ9Vδ2 T‐cells are universally present in humans and represent one of the most prevalent TCR reactivities, evolutionarily conserved across diverse mammalian species. They are an innate‐like subset featuring a semi‐invariant TCR repertoire that drives their well‐recognized reactivity to small, non‐peptidic phosphoantigens (pAg). Crucially, they can distinguish between highly immunostimulatory microbially derived pAg and much less potent host‐derived pAg, with the former effectively acting as a pathogen associated molecular pattern (PAMP) and the Vγ9Vδ2 TCR as a surrogate pattern recognition receptor (PRR). Ample evidence supports important Vγ9Vδ2‐mediated contributions to immunity against diverse pathogenic bacteria and parasites, mediated by their potent effector and immunoregulatory functions. The molecular basis of the pAg sensing mechanism underpinning such responses has, however, remained highly mysterious. Despite this, past studies have established that pAg sensing is MHC‐independent, extremely fast, exquisitely pAg‐sensitive, and dependent upon target cell expression of key BTN‐family molecules, notably BTN3A and BTN2A1. Here we contextualize these findings and several recent studies addressing pAg sensing. We integrate these into a single unified theory of pAg sensing interpretable from different perspectives, both intracellular and extracellular, biophysical, and topological. We prioritize critical questions to address in the context of this proposed model. Finally, we suggest the model will provide a molecular template for antigen recognition by other related γδ T‐cell subsets.

## 1 Introduction

Since their serendipitous discovery in 1986 [1], γδ T cells, which have been retained throughout ~450–500 M years of vertebrate evolution [2], have posed immunologists major challenges, particularly regarding the antigens they recognize and respond to. The γδ T cell receptor (TCR) that both defines the compartment and underpins their recognition capabilities is structurally analogous to the αβ TCR and to the Fab region of antibodies. Each γδ TCR chain incorporates two immunoglobulin (Ig) variable domains, with complementarity determining regions (CDRs) positioned at the membrane‐distal tip of the TCR, equivalent to those that make contacts with antigen (in the case of antibodies) or peptide–MHC (pMHC) complexes in the case of αβ TCRs. However, early studies suggested that γδ T cell antigens were unlikely to include the class I and class II MHC molecules that can stimulate most CD8 and CD4 T cells.

A major step forward in understanding at least some of the antigenic targets of γδ T cells was provided by initial observations centered on Vγ9Vδ2 T cells. This distinctive γδ T cell subset, universally present in healthy human blood, typically comprises the majority of circulating γδ T cells, which themselves typically represent < 10% of all T cells [3]. However, in a range of human infections, Vγ9Vδ2 T cells were found to expand to high levels, even increasing to a majority of the entire T cell compartment in some cases. Such expansions have now been observed for infections with bacterial species (e.g., S*almonella* *spp*., *Brucella melitensis* , *Francisella tularensis* ), mycobacteria (e.g., *Mycobacterium tuberculosis* [MTb]) and protozoan parasites (e.g., *Plasmodium vivax* or *Plasmodium falciparum*) [3].

**FIGURE 1:**
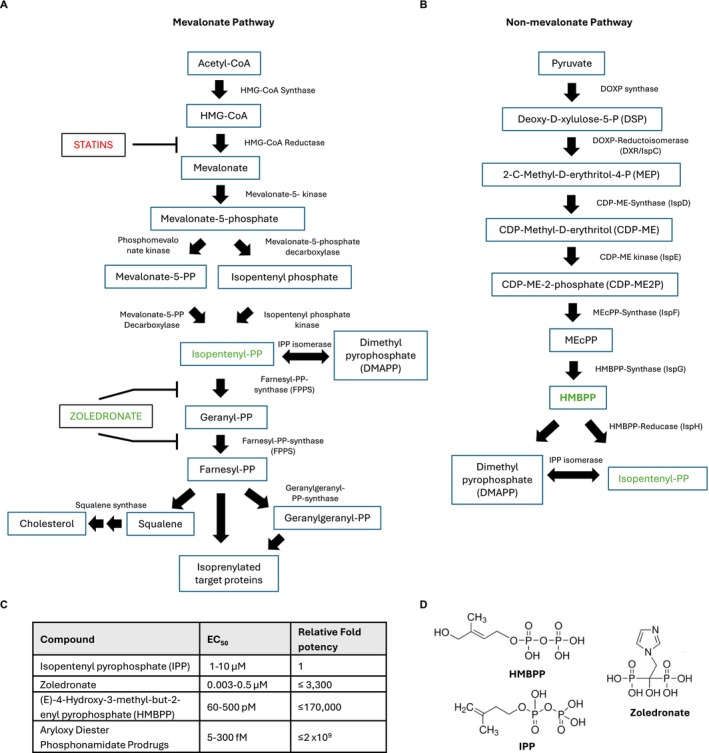
Metabolic pathways involved in pAg generation. (A) The mevalonate pathway utlilsed by eukaryotes, archaea and some bacteria, which enables production of cholesterol and underpins isoprenylation of cellular proteins crucial for a range of cellular functions, with inhibition by Statins (red) and promotion of IPP accumulation by Zoledronate (green) indicated. (B) The non‐mevalonate or 2‐C‐methyl‐D‐erythritol 4‐phosphate (MEP) pathway, as utilized by many gram‐negative bacteria, which generates precursors for isoprenoid biosynthesis, including HMBPP (green), a potent stimulator of Vγ9Vδ2 T cells. (C) Relative potency of naturally occurring (HMBPP, IPP) phosphoantigens (pAg), pharmacological stimulators of the IPP accumulation (Zoledronate), and pAg prodrugs for Vγ9Vδ2 activation are shown. (D) Chemical structures of HMBPP, IPP and Zoledronate, as indicated.

Seminal early studies established that soluble extracts from several such pathogens, such as mycobacteria [4, 5, 6], were also able to stimulate and/or expand Vγ9Vδ2 T cells in vitro. The active compounds were shown to be small, protease‐resistant (and therefore non‐peptidic), pyrophosphate‐containing compounds, with the pyrophosphate moiety critical for stimulatory activity [7, 8, 9, 10, 11]. Subsequent studies identified one such pathogen‐derived stimulatory compound, albeit one with a relatively low potency for Vγ9Vδ2 activation, as isopentenyl pyrophosphate (IPP) [10]. However, intriguingly, IPP, a central intermediate in the mevalonate pathway of isoprenoid biosynthesis (Figure 1A), was found to be present both in pathogen extracts and in human cell lines, and pathogens such as mycobacteria were observed to produce not just IPP but a range of similar phosphate‐containing antigens. A clue to their identity was provided by studies revealing a second pathway of IPP biosynthesis (the non‐mevalonate or 2‐C‐methyl‐D‐erythritol 4‐phosphate (MEP pathway) restricted to bacteria and plants. Building on experiments correlating pathogen use of the MEP pathway with capacity to stimulate Vγ9Vδ2 T cells [12, 13], subsequent studies deleting enzymes in the pathway clarified that the key antigenic metabolite was a pyrophosphate termed (E)‐4‐hydroxy‐3‐methyl‐but‐2‐enyl pyrophosphate (HMBPP) [14]. Crucially, this antigenic metabolite, derived from the non‐mevalonate or MEP pathway of isoprenoid biosynthesis absent in human cells (Figure 1B), displayed a potency for Vγ9Vδ2 activation orders of magnitude (> 10,000) higher than that of the chemically similar IPP molecule also present in human cells [15, 16, 17] (Figure 1C).

The studies above established that the recognition of small, non‐peptidic phosphate‐containing compounds, or phosphoantigens (pAg), is central to the immunobiology of Vγ9Vδ2 T cells. The finding that HMBPP and IPPhad such different potency for Vγ9Vδ2 T cell stimulation (Figure 1C), despite close chemical similarity (Figure 1D), confirmed earlier suspicions that a mechanism likely exists whereby Vγ9Vδ2 T cells discriminate pyrophosphate antigens of foreign origin from those derived from self [3]. In addition, it bolsters the case that HMBPP can be viewed as a *bona fide* PAMP [18], not least because HMBPP is a critical intermediate in the generation of isoprenoid compounds that underpin bacterial cell wall biosynthesis [3]. Moreover, consistent with pAg stimulation being restricted to Vγ9Vδ2 T cells, subsequent studies have also shown that the ability to sense pAg is TCR‐dependent [3, 19, 20]. Vγ9Vδ2 T cells therefore represent an interesting case of the somatically recombined TCR, often thought of as a hallmark of adaptive immunity, acting like a surrogate PRR, thereby mimicking the innate immune system.

The concept that Vγ9Vδ2 T cells may in some sense be ‘innate‐like’ is supported by several other observations that distinguish them from both conventional adaptive αβ T cells and also adaptive‐like γδ T cells [3, 21]. A stark difference is the polyclonal and near 100% reactivity of Vγ9Vδ2 T cells to pAg [3, 21], which contrasts with adaptive compartments, which typically exhibit diverse antigen reactivities that are each highly clonotypically restricted.

A second consideration is that in adult humans, Vγ9Vδ2 T cells largely lack a phenotypically naïve component, instead adopting a range of effector phenotypes from very early in life [21, 22], expressing perforin, granzyme, displaying potential for cytokine production, and demonstrating an ‘innate‐effector’ transcriptional profile featuring expression of genes such as PLZF, which is common to other innate‐like populations such as MAITs and iNKTs [23]. Furthermore, the TCR repertoire of Vγ9Vδ2 T cells is highly distinct from that of adaptive subsets, typically featuring a Vγ9 chain using JgP and displaying limited CDR3 diversity, paired with a Vδ2 chain of higher diversity but constrained CDR3 length relative to Vδ1 [24, 25]. The limited TCR‐Vγ diversity, in particular, is reminiscent of TCRα sequences in MAIT and iNKT cells and justifies the Vγ9Vδ2 repertoire as being ‘semi‐invariant’ [18, 24].

Moreover, thymic development of Vγ9Vδ2 T cells demonstrates some stark differences to adaptive‐like immune subsets. Vγ9Vδ2 T cells are some of the earliest T cells to develop in the fetal thymus during early gestation, such that Vγ9Vδ2 T cells are the prevalent γδ T cell by mid‐gestation, after which a wave of Vδ1 production occurs alongside αβ T cell development [24, 25]. A fifth feature of Vγ9Vδ2 T cells that differs markedly from conventional adaptive T cell subsets is their reasonably uniform post‐natal development into a sizeable effector population. In most individuals, such Vγ9Vδ2 innate‐effector T cells expand in peripheral blood over the first 3 years of life to become the dominant γδ T cell subset [24]. Although this was previously thought to reflect pAg‐driven Vγ9Vδ2 expansion in response to bacterial exposure in early life, several lines of evidence, including changes in Jd3 vs. Jd2 usage between cord and adult blood [24], Vγ9Vδ2 reconstitution following SCT [26, 27, 28], and direct studies on thymus [29], favor ongoing thymic production of Vγ9Vδ2 T cells after birth. In addition, recent studies indicate upregulation of Tbet and Eomes and Granzyme expression on Vγ9Vδ2 T cells before they exit the thymus [30], consistent with previous phenotypic analysis of cord blood Vγ9Vδ2 T cells indicating evidence of early effector marker expression [21]. Collectively, these data suggest the likelihood of ongoing production of pre‐committed innate‐like, ‘effector‐ready’ Vγ9Vδ2 T cells from early life onwards, in stark contrast to adaptive T cell compartments [18].

The above evidence points to an important role for Vγ9Vδ2 T cells in anti‐microbial immunity from early life onwards. Consistent with this, the functional response induced downstream of pAg recognition includes cytokine production, cytotoxicity, and changes in chemokine receptor expression (e.g., CCR5, CXCR3), facilitating transendothelial migration and trafficking of activated Vγ9Vδ2 cells to sites of inflammation, including in response to infection [31]. Moreover, studies in primates support a role for Vγ9Vδ2 T cells as multifunctional effectors of immune protection against Mtb infection [32, 33, 34, 35, 36]. These have led to studies employing a promising approach based on vaccination with attenuated HMBPP‐producing Listeria, which boosted Th1‐like Vγ9Vδ2 T cell subsets in the airway and led to containment of tuberculosis infection after pulmonary challenge [37]. This protection was likely based on an exacerbated, fast‐acting Vγ9Vδ2 response involving both IFNγ and perforin, inhibiting MtB growth and facilitating earlier pulmonary CD4 and CD8 responses to tuberculosis challenge [37].

In addition to HMBPP‐stimulated antimicrobial immunity, Vγ9Vδ2 T cells are also able to recognize and mount responses (including cytotoxicity and cytokine production) to human cancer cells based on increased flux through the mevalonate pathway that results in elevated levels of IPP [38]. Moreover, this axis of recognition can be boosted by small molecule drugs, since clinically approved aminobisphosphonates (NBP) such as Zoledronate (Figure 1D) have been shown to sensitize target cells to recognition by Vγ9Vδ2 T cells and also expand them both in vitro and in vivo, via a mechanism that involves boosting of IPP levels via inhibition of farnesyl pyrophosphate synthase (FPPS), the enzyme that catabolizes IPP [3, 39] (Figure 1A). Alongside their prevalence in peripheral blood and potential to mediate MHC‐unrestricted targeting of diverse cancer cells, this pharmacological manipulability has helped stimulate major efforts to exploit Vγ9Vδ2 T cells for cancer immunotherapy [40].

Thus, a substantial body of evidence supports the importance and potential for therapeutic exploitation of the Vγ9Vδ2 T cell subset in the context of both infection and cancer.

Given their important contribution to antimicrobial immune defense and potentially anti‐tumor immunity, the evolutionary conservation of Vγ9Vδ2 T cells across the vertebrate lineage is of interest. Although Vγ9Vδ2 T cells are absent in mice and were thought to be restricted to primates, recent studies have reported the presence of equivalent T cells in Alpaca [41], indicating that this system of TCR‐mediated pAg sensing likely arose and is retained in a substantial portion of placental mammals.

In summary, the immunobiology of Vγ9Vδ2 T cells, undoubtedly one of the most prevalent T cell antigen reactivities in humans, appears to be radically distinct from adaptive T cell subsets [18] and is centrally focused on their ability to sense and respond to the presence of dysregulated pAg. However, the molecular mechanism underlying this remarkable antigen recognition capability, which clearly differs so radically from αβ T cell recognition, has remained both highly mysterious and a subject of intense interest. In this review, we attempt to summarize and integrate diverse research findings, including recent advances that shed light on the molecular mechanisms involved and inform mechanistic models underpinning this pAg‐sensing process.

## 2 Mechanistic Pillars

### 2.1 Core Observations

Several core observations regarding Vγ9Vδ2 T cell phosphoantigen (pAg) sensing are worthy of note before considering underlying molecular mechanisms. Firstly, as noted above, early studies demonstrated that T cell pAg sensing is entirely dependent on the Vγ9Vδ2 TCR, since it could be conferred by transduction of Vγ9Vδ2 TCRs into recipient T cell lines [20], and also depends on cell–cell contact [42]. Furthermore, studies established that both TCRγ and TCRδ chains are essential to the process. Thus, T cell recognition of pAg is inherently encoded within the Vγ9Vδ2 TCR itself. Secondly, the kinetics of the pAg sensing process are extremely fast. In in vitro *assays* monitoring Ca^2+^ flux, activatory signaling in the T cell is initiated within seconds of target cell exposure to pAg [42]. This suggested that pAg may not require extensive processing to facilitate the recognition process, in stark contrast to the processing of intact protein antigen destined for cell surface presentation as MHC‐bound peptides, which can take up to 60 min. A third observation was that human Vγ9Vδ2 T cells are restricted to recognition of pAg exposure on human cells, suggesting the presence of host‐encoded factors in target cells that underpin pAg‐proximal sensing events [43]. Finally, Vγ9Vδ2 T cells are exquisitely sensitive to the presence of foreign pAg, emphasized by their potent stimulation by the canonical microbial pAg, (E)‐4‐Hydroxy‐3‐methyl‐but‐2‐enyl pyrophosphate (HMBPP). HMBPP, an intermediate in the non‐mevalonate or MEP pathway underpinning isoprenoid biosynthesis that is absent in humans but utilized by many pathogenic bacteria, has an EC_50_ in the low nM range [10, 17] (Figure 1). In contrast, the potency of the host‐derived isopentenyl pyrophosphate (IPP), derived from the mammalian mevalonate pathway, is > 10,000 times less potent, despite its structural similarity to HMBPP [10, 17] (Figure 1). This potential for such high sensitivity and specificity for foreign pAg mirrors that of conventional αβ T cells for foreign peptide presented by self‐MHC, and suggests that broadly analogous TCR triggering and signaling processes may be initiated downstream of Vγ9Vδ2 TCR‐mediated antigen recognition. Collectively, these features point to Vγ9Vδ2 T cell pAg sensing being a highly evolved system, radically distinct from TCR/pMHC recognition in terms of molecular events, that links rapid detection of foreign pAg in target cells to recognition of a cognate antigen capable of triggering the Vγ9Vδ2 TCR.

### 2.2 Central Players and Their Molecular and Structural Roles

#### 2.2.1 The Vγ9Vδ2 TCR

The molecular features of the Vγ9Vδ2 TCR, the signature characteristic of the Vγ9Vδ2 T cell subset, underpin the latter's polyclonal and near‐universal reactivity to pAg [3, 21]. At the DNA level, deep sequencing studies have highlighted a semi‐invariant Vγ9Vδ2 TCR repertoire, involving the prevalence of public Vγ9‐JyP TCR sequences generated by both recurrent rearrangements and synonymous recombination events, and featuring limited non‐templated (N)/palindromic (P) region nucleotide addition [24]. In adults, Vδ2 CDR3 sequences typically utilize Jδ1, while Jδ3 and Jδ2 are more frequently observed in cord blood [21, 24]. Vδ2 chain sequences encode a crucial hydrophobic residue, typically Val/Leu/Ile, at position 5 of the CDR3, which is essential for pAg recognition [3]. Such features underscore the conserved, innate‐like biology of the Vγ9Vδ2 T cell subset, and contrast markedly with TCR repertoire features of adaptive‐like γδ T cell populations. Indeed, comparative analyses indicate that CDR3 lengths of Vγ9Vδ2 TCRs are significantly constrained relative to the highly diverse CDR3 repertoire of Vδ2^neg^ TCRs [44]. This likely relates to structural constraints on Vγ9Vδ2 TCR recognition of a conserved ligand(s), whereas extreme CDR3 diversity in Vδ2^neg^ TCRs likely reflects a diverse range of potential ligands. Consistent with this hypothesis, mutagenesis studies by Morita supported the idea that all CDRs of the Vγ9Vδ2 TCR are involved in pAg recognition [44].

**FIGURE 2:**
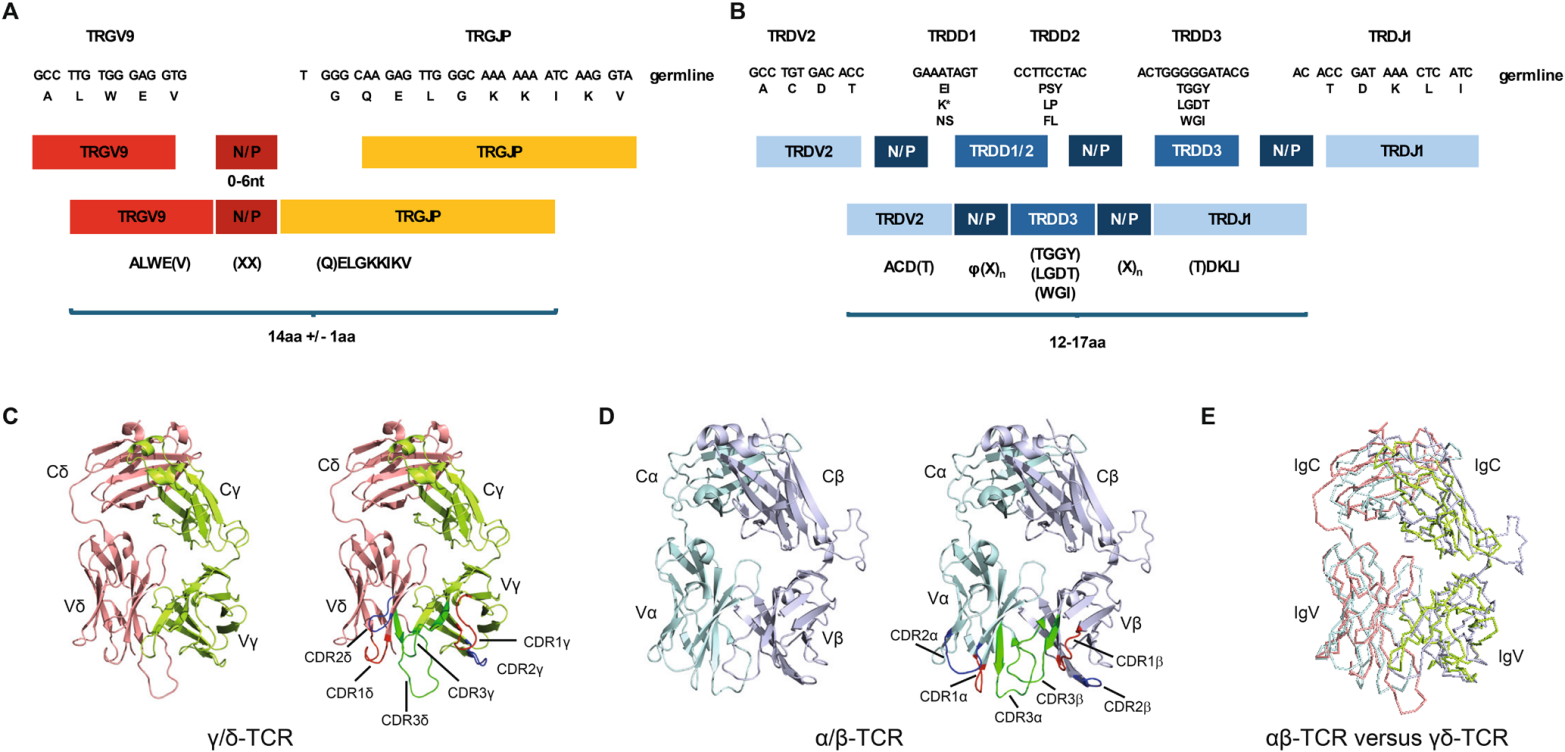
Recombination and structure of the Vγ9Vδ2 TCR. (A) TCR recombination in Vγ9Vδ2 T cells. To generate the Vγ9 chain, the TRGV9 gene segment is joined to the TRGJP gene segment, following exonuclease activity which can remove one or more nucleotides at the ends of the gene segments (resulting in potential loss of the amino acids in brackets), and N‐nucleotide addition by TdT (0–8 nt, average ~ 2), resulting in the addition of one or more amino acids (X). The resulting CDR3 sequence is typically 13–15 amino acids, with an average of 14 amino acids. (B) The Vδ2 chain is generated by joining of TRDV2 to at least one TRDD gene segment (typically TRDD3), which can be used in any of the three reading frames, and which is typically joined to TRDJ1 in postnatally derived Vγ9Vδ2 T cells. In some cases a second TRDD gene segment (TRDD1 or TRDD2) is used in addition to TRDD3. As with the Vγ9 chain, exonuclease activity can remove nucleotides at the end of TRDV2, TRDJ1 and any of the TRDD gene segments used, and TdT adds N nucleotides at any of the joins (0–15 nt across the CDR3, average ~8 nt), resulting in a CDR3 length of 12–17 amino acids. A hydrophobic amino acid (φ) is typically found at CDR3 position 5, which is derived from N nucleotides or the TRDD gene segment and is critical for pAg sensing. (C‐E) Comparison of γδ‐TCR and αβ‐TCR ectodomain structures. (C) Crystal structure of the γδ‐TCR ectodomain (PDB ID: 1HXM) (left panel), with complementarity‐determining regions (CDRs) highlighted (right panel). (D) Crystal structure of the αβ‐TCR ectodomain (PDB ID: 3REV) (left panel), with CDR regions highlighted (right panel). (E) Superimposition of the γδ‐TCR onto the αβ‐TCR demonstrates reveals a similar overall structure. TCR are colored as shown in panels C and D.

The first structural data on the Vγ9Vδ2 TCR noted a positively charged surface patch hypothesized at the time to be involved in direct binding of pAg [45]. Although pAg have subsequently been shown to act intracellularly [46], this study was important in defining an overall similar architecture to the αβ TCR (Figure 2), while also highlighting notable differences within the γδ TCR constant regions. Recent cryogenic‐electron microscopy (cryo‐EM) studies have enabled visualization of the γδ TCR in the context of CD3 components [47, 48]. One study highlighted that, in contrast to some Vδ2^neg^ TCRs, the Vγ9Vδ2 TCR is monomeric, with limited potential for flexibility relative to Vδ2^neg^ TCRs [48].

Finally, despite the polyclonal nature of recognition, two noteworthy features of Vγ9Vδ2 TCR biology merit further attention. Firstly, TCR‐intrinsic clonotypic differences have been noted that affect the sensitivity of pAg recognition [49]. Although incompletely understood, this is consistent with studies which emphasize the impact of CDR3γ and CDR3δ on recognition [50], and likely reflects a degree of plasticity/redundancy at the population level in the recognition of BTN‐family ligands. Secondly, while Vγ9Vδ2 TCR repertoires typically demonstrate oligoclonality, rare, highly focused expansions of individual Vγ9Vδ2 clonotypes are sometimes observed [21, 24], the significance of which is currently unclear.

### 2.3 BTN Family Molecules

#### 2.3.1 The BTN Family

**FIGURE 3:**
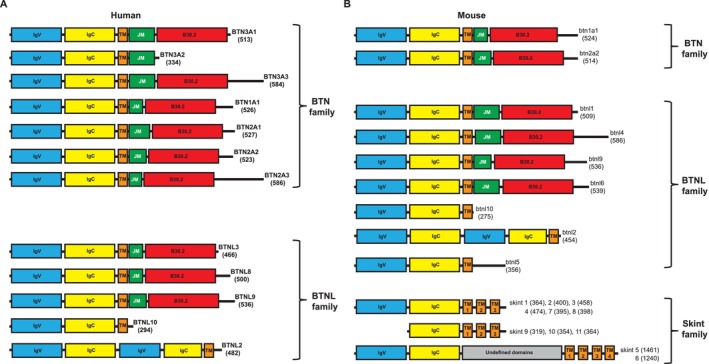
Domain architecture of BTN family members. (A) The human BTN family encompasses 7 BTN proteins (BTN1A1, BTN2A1, BTN2A2, BTN2A3 (pseudogene), BTN3A1, BTN3A2, BTN3A3), 5 butyrophilin‐like (BTNL) proteins (BTNL2, BTNL3, BTNL8, BTNL9, BTNL10 (pseudogene)) and the selection and upkeep of intraepithelial T‐cells‐like (SKINTL) factor (pseudogene, not shown). (B) In mice 21 BTN‐related proteins have been described: Btn1a1, Btn1a2, Btnl1, Btnl2, Btnl4, Btnl5, Btnl6, Btnl7 (pseudogene), Btnl9, Btnl10 and Skint1‐11. Skint 5 and 6 encompass additional undefined regions in their ectodomain. Numbers in parenthesis indicate amino acid length of each protein. Abbreviations: IgV (immunoglobulin variable domain), IgC (immunoglobulin constant domain), TM (transmembrane region), JM (juxtamembrane region) and B30.2 (SPRY domain).

Butyrophilins (BTN) are a family of type I transmembrane glycoproteins collectively expressed in a diverse range of cell types and that play a crucial role in regulating T cell activation and differentiation. Their name, literally meaning ‘butter‐loving’, originates from the prototypic member, BTN (the human orthologue of which is BTN1A1), which is highly expressed in mammary gland epithelium during lactation [51] and both associates with and regulates milk lipid droplet formation during secretion [51, 52]. In humans, the BTN family encompasses 7 BTN proteins (BTN1A1, BTN2A1, BTN2A2, BTN2A3, BTN3A1, BTN3A2, BTN3A3), 5 butyrophilin‐like (BTNL) proteins (BTNL2, BTNL3, BTNL8, BTNL9, BTNL10) and the selection and upkeep of intraepithelial T‐cells‐like (SKINTL) factor [53] (Figure 3A). Of note, BTN2A3, BTNL10 and SKINTL are pseudogenes. In mice, 21 BTN‐related proteins have been described: Btn1a1, Btn1a2, Btnl1, Btnl2, Btnl4, Btnl5, Btnl6, Btnl7, Btnl9, Btnl10, and Skint1‐11, demonstrating notable species‐specific differences [53] (Figure 3B). BTN/BTNL proteins belong to the B7 superfamily, which also includes co‐receptor proteins CD80, CD86, ICOSL, and PD‐L1. Structurally, BTN family members typically feature two extracellular immunoglobulin (Ig) domains (IgV and IgC), a transmembrane region, an intracellular juxtamembrane coiled‐coil domain followed by a variable intracytoplasmic tail that includes a B30.2 (PRYSPRY) module [53] (Figure 3).

Beyond the role of certain members in regulating γδ T cells, BTN family members play roles in the regulation of αβ T cell responses, exhibiting both co‐stimulatory and co‐inhibitory functions depending on the specific context and experimental conditions [54]. BTN proteins, expressed in various immune and epithelial cells, interact with receptors on opposing cells, an example being BTN2A1 interaction with DC‐SIGN on dendritic cells [55]. Although many specific receptors remain unidentified, notably, BTNL8 has been shown to co‐stimulate CD4 and CD8 T cell proliferation and cytokine production [56], while BTN3A engagement can either co‐stimulate or inhibit T cell activation depending on the specific monoclonal antibody used [57, 58]. BTN2A2 and BTNL2 also demonstrate inhibitory effects on T cell activation and promote the induction of regulatory T cells (Tregs), suggesting a multifaceted role in modulating immune responses [59, 60]. Furthermore, BTN and BTNL proteins are implicated in maintaining intestinal immune homeostasis and have been associated with inflammatory bowel diseases, indicating their importance in local immune regulation [61].

As outlined below, structurally, the BTN ectodomain can form V‐shaped or head‐to‐tail dimers, with the coiled coil and juxtamembrane regions domains potentially regulating dimer pairing, trafficking, and conformation of homomeric and heteromeric BTN complexes. The B30.2 domain, a hallmark of BTN family members, mediates diverse protein–protein interactions, particularly in immune signaling and regulation [62, 63]. It serves as a protein‐interaction module, with its specific binding partners and determinants of specificity the focus of ongoing study. Generally, BTNL proteins exhibit a similar structure to BTNs, whereas Skint members are characterized by three transmembrane passes and typically lack the B30.2 domain and certain Ig domains [53, 64]. This structural variation may reflect differing functional roles and regulatory mechanisms within the immune system.

#### 2.3.2 BTN3A

Building on studies demonstrating the importance of the B7‐like molecule Skint‐1 in the selection and responses of mouse dendritic epidermal T cells (DETC) [65, 66, 67], Harly et al. investigated the relevance of the BTN family, the proteins with the greatest similarity in humans, to human γδ T cells. BTN3A emerged from these studies as playing a critical role [68] in promoting Vγ9Vδ2‐mediated T cell activation. Notably, the authors also established an essential, non‐redundant role for the BTN3A1‐B30.2 domain in both pAg sensing and response to aminobisphosphonate drugs [68], consistent with a role for BTN3A1 as the chief ‘pAg sensor’. In addition, antibodies to BTN3A IgV‐like domains could either trigger (20.1) or block (103.2) activation, suggesting the importance of the extracellular domain of BTN3A proteins in activation [68]. Interestingly, BTN3A2 and BTN3A3 were found to be permissive for 20.1‐mediated activation, but not for pAg/aminobisphosphonate‐mediated activation in the absence of BTN3A1 expression [68].

**FIGURE 4:**
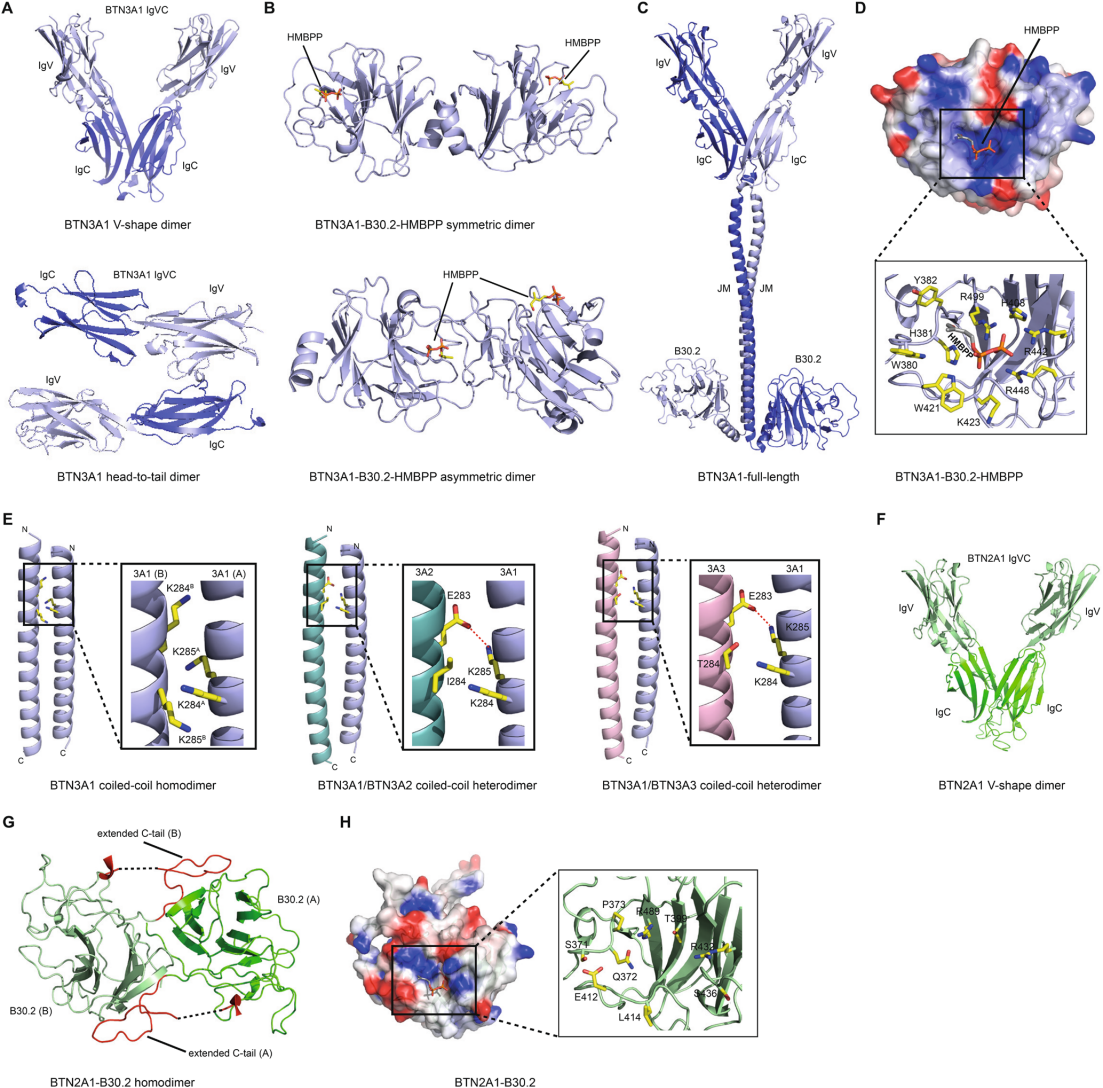
Structural Insights into BTN3A1 and BTN2A1: Conformational States, Dimerization Interfaces, and Electrostatic Properties. (A) BTN3A1 V‐shape (top panel) and head‐to‐tail dimer (bottom panel) states observed within the crystal lattice (PDB ID: 4F80). (B) Structural arrangements of BTN3A1‐B30.2 monomers (PDB ID: 5ZXK), showcasing a symmetric dimer interface where two HMBPP molecules are positioned distally (top panel), and an asymmetric dimer interface featuring one HMBPP molecule in proximity to the dimer interface (bottom panel). (C) AlphaFold2‐derived molecular model of the full‐length BTN3A1 homodimer. The JM regions form a coiled‐coil and serve as a linker between the ectodomain and intracellular B30.2 domains. (D) Electrostatic potential surface of the BTN3A1 B30.2 domain (red, negative charge; blue, positive charge) (PDB ID: 5ZXK). The HMBPP binding site is highlighted, accompanied by an expanded view of key BTN3A1‐B30.2 residues that contribute to the HMBPP binding site. (E) Models of the BTN3A1 coiled‐coil homodimer (left panel), BTN3A1‐BTN3A2 coiled‐coil heterodimer (middle panel) and BTN3A1‐BTN3A3 coiled‐coil heterodimer (right panel) generated using CCBuilder2 (residues Q273‐L312 were used for coiled‐coil models). Dimer interface residues at positions 283–285 are shown as ball and stick. Polar interactions are highlighted (red dashed lines). Each monomer within the homodimer has been labeled A or B. (F) AlphaFold2‐derived molecular model of the BTN2A1‐V‐shape dimer. (G) Crystal structure of BTN2A1‐B30.2 homodimer (PDB ID: 8IGT). The dimer interface is predominantly stabilized by an extended C‐terminal tail (red), which is notably absent in BTN3A1. (H) Electrostatic potential surface of the BTN2A1‐B30.2 domain (red, negative charge; blue, positive charge). This domain is characterized by the absence of the positive groove essential for HMBPP binding, highlighting a significant structural difference compared to BTN3A1.

Structural analyses of BTN3A ectodomains have shown that BTN3A molecules can adopt two alternative dimer configurations, referred to as ‘V‐shaped’ and ‘head‐to‐tail’ dimer forms [69] (Figure 4A). The V‐shaped dimer is formed by extensive symmetrical intermolecular contacts between membrane‐distal C‐like domains. In contrast, the head‐to‐tail dimer features a non‐symmetrical interface in which the IgV domain from one BTN3A associates with the IgC domain from a second BTN3A [69]. Similarly, intracellular B30.2 domain crystal structures indicate a propensity for dimer formation [70] (Figure 4B). Finally, the intracellular alpha helical regions of BTN3A molecules are predicted to form dimeric coiled‐coils [71] (Figure 4C). Collectively, these data indicate BTN3A molecules are highly likely to be present at the cell surface as obligate dimers, a conclusion supported by the detection of BTN3A homo‐BRET signals that are unaltered throughout the pAg sensing process [72].

The molecular events underlying proximal pAg detection by BTN3A1 have been the subject of some controversy. Vavassori et al. reported that the BTN3A1 IgV domain could present pAg antigens, enhancing Vγ9Vδ2 TCR binding to BTN3A1, based on crystal structures of the BTN3A1 IgV domain and surface plasmon resonance [73]. However, subsequent seminal work of Sandstrom et al. conclusively refuted these claims, demonstrating instead that pAg were bound directly by the intracellular B30.2 domain of BTN3A1 [46]. Their study included a crystal structure of the B30.2 domain, which identified a positively charged pocket strongly implicated in pAg binding, based partly on isothermal titration calorimetry (ITC) data [46] (Figure 4D). Subsequent corroboration of pAg binding to the BTN3A1‐B30.2 domain has been provided by multiple groups using various techniques, including ITC, nuclear magnetic resonance spectroscopy, x‐ray crystallography, and fluorescence polarisation [74, 75, 76, 77]. In addition, tangential support for intracellular pAg sensing comes from the development of pAg prodrugs specifically engineered for enhanced intracellular bioavailability, some of which display > 10,000‐fold increased potency compared to HMBPP [78, 79].

Although BTN3A1 is essential for pAg sensing, several studies have emphasized that in humans, co‐expression of either BTN3A2 or BTN3A3 synergizes with BTN3A1 to greatly boost the efficiency of pAg sensing [76, 80, 81, 82]. This synergy may partly be attributed to a chaperone‐like function of BTN3A2/A3 in boosting cell surface expression of BTN3A1 [82]. This effect appears to be governed partly by a negatively charged triplet‐amino acid stretch in the BTN3 transmembrane (TM) domain that preferentially drives the formation of BTN3A1/A2 and BTN3A1/A3 heterodimers that traffic efficiently to the cell surface, and destabilizes BTN3A1 homodimers that don't [81] (Figure 4E). However, evidence also suggests that in the context of BTN3A1/A2 or BTN3A1/A3 heterodimers, the IgV domain of BTN3A2/A3 is functionally more important than the BTN3A1 IgV [81]. Of note, some mammalian species only possess one BTN3 molecule, which closely resembles BTN3A3 but, unlike human BTN3A3, contains a functional B30.2 domain, potentially resembling a primordial BTN3 protein [41].

#### 2.3.3 BTN2A1

Despite the importance of BTN3A in pAg sensing, data indicated that a host‐encoded gene(s) on chromosome 6, alongside BTN3A1/A2/A3, was crucial in the process, leading to a search for the critical ‘Factor X’ [83, 84]. Two separate approaches, one based on radiation hybrids [85] and the other on clustered regularly interspaced short palindromic repeats (CRISPR) technology [86], ultimately led to the identification of the same gene, BTN2A1, as fulfilling this role. Importantly, both studies demonstrated that BTN2A1 exhibited potent functional synergy with BTN3A1 in enhancing pAg sensing [85, 86]. Retrospective analyses from previous studies indicate that BTN2A1 is dispensable for 20.1 mAb‐mediated sensitization of BTN3A‐positive cells.

Modeling analyses predicted BTN2A1 forms a V‐shaped dimer stabilized by substantial non‐covalent IgC‐IgC interactions, as for BTN3A molecules [85] (Figure 4F). This was corroborated by sodium dodecyl sulfate–polyacrylamide gel electrophoresis (SDS‐PAGE) analysis, which established that BTN2A1 homodimers can be further stabilized by an additional interchain disulphide bond [85]. Recent crystallographic studies of the BTN2A1 ectodomain confirmed these findings [87], and homo‐Bioluminescence Resonance Energy Transfer (BRET) experiments are consistent with BTN2A1 forming a dimer at the cell surface [72]. Of note, BTN2A1 contains an intracellular B30.2 domain that is also thought to be dimeric based on crystallographic and size‐exclusion chromatography coupled with multi‐angle light scattering (SEC‐MALS) data [88] (Figure 4G). The crystal structure confirmed that BTN2A1‐B30.2 lacks the basic pocket present in BTN3A1‐B30.2 that mediates HMBPP binding (Figure 4H), explaining the inability of the BTN2A1‐B30.2 domain to independently bind pAgs [72, 86].

### 2.4 Intermolecular Interactions—Evidence and Controversy

Here we outline our current understanding of the intermolecular interactions involving some of the key players outlined above, including BTN3A, BTN2A1, and pAg themselves, which provide a basis for mechanistic models of the pAg‐sensing process.

#### 2.4.1 TCR Recognition of the BTN2A1 Ectodomain

**FIGURE 5:**
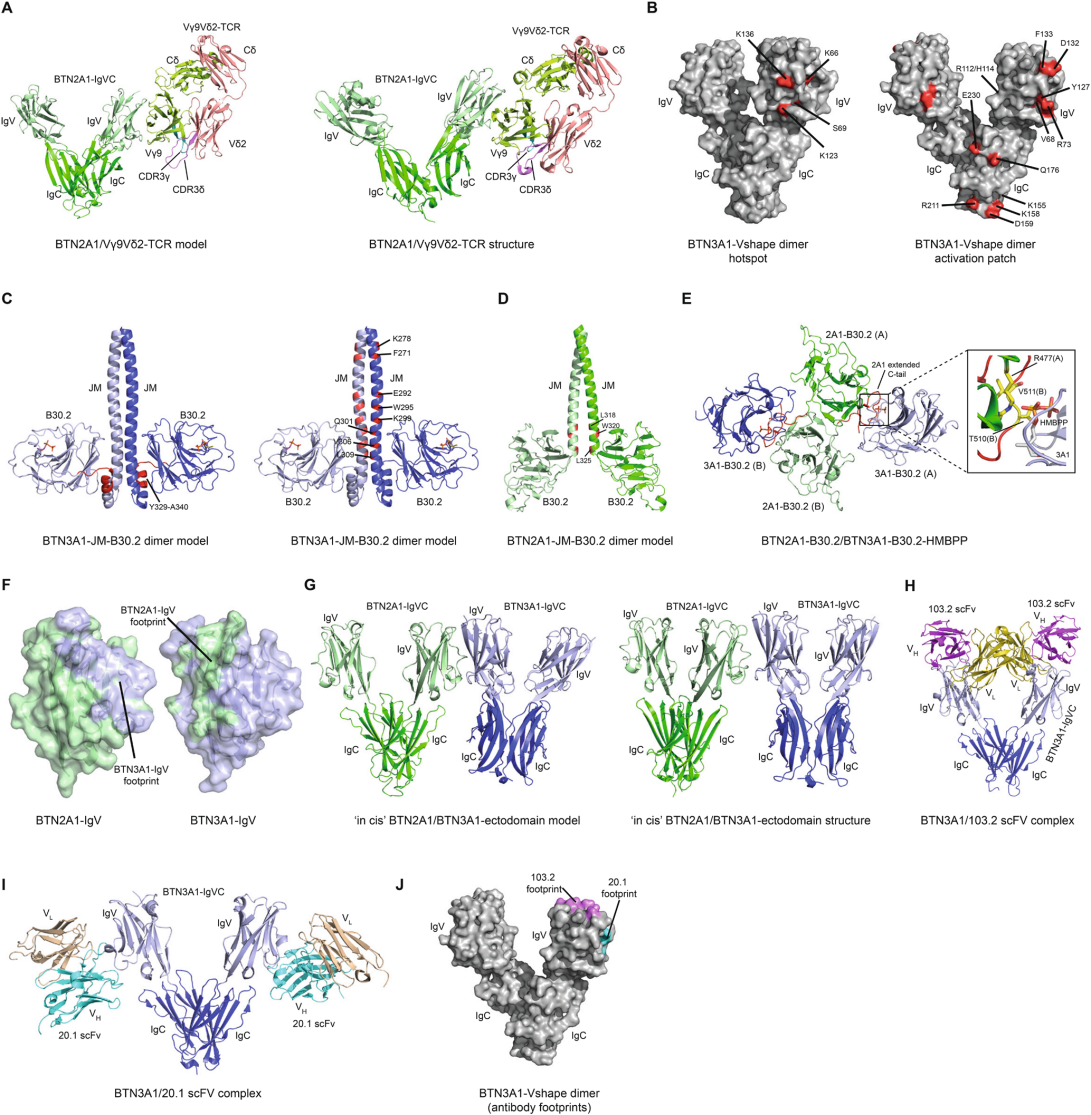
Molecular Basis of BTN2A1 and BTN3A Interactions with Vγ9Vδ2 TCR. (A) NMR‐informed model of the BTN2A1‐ectodomain/Vγ9Vδ2‐TCR complex (left panel) [89]. Crystal structure of the BTN2A1‐ectodomain/Vγ9Vδ2‐TCR complex (PDB ID: 8DFW, right panel) [87]. (B) Molecular surface representation of the BTN3A1‐ectodomain V‐shape dimer. Residues identified by Willcox et al. [89] that impact phosphoantigen sensing by MOP‐TCR are highlighted (red, left panel). Residues identified by Fulford et al. [87] that led to an abrogation of zoledronate‐dependent Vδ2^+^ γδ T cell activation are highlighted (red; right panel). (C) AlphaFold2‐derived molecular model of the BTN3A1‐JM‐B30.2 dimer. Regions identified by Peigne at al 2017 that impact activation of Vγ9Vδ2 T cells are shown (red), specifically JM residues located near the B30.2 domain (left panel) [91]. Wang et al. [71] identified coiled‐coil regions critical for activation of Vγ9Vδ2 T cells (right panel). (D) AlphaFold2‐derived molecular model of the BTN2A1‐JM‐B30.2 dimer. Hsiao et al. [72] identified residues within the BTN2A1 JM region as important for Vγ9Vδ2 T cell phosphoantigen response (red). (E) Structure of the BTN3A1‐B30.2‐HMBPP/BTN2A1‐B30.2 complex (PDB ID: 8JYE). HMBPP functions as a molecular glue promoting BTN2A1‐B30.2 and BTN3A1‐B30.2 association. Magnified views of the interactions between the BTN2A1 B30.2 dimer and the HMBPP–BTN3A1 B30.2 are shown. Extended BTN2A1 C‐terminal tail plays a critical role in mediating interactions with HMBBPP. (F) Molecular surface representation of the BTN2A1‐IgV (green) and BTN3A1‐IgV (blue) domains. The putative binding footprints are highlighted on each surface. (G) Full ‘in cis’ V‐shaped BTN3A1‐BTN2A1 ectodomain interaction extrapolated from the BTN2A1‐IgV/BTN3A1‐IgV model (left panel) [89]. Crystal structure of the ‘in cis’ BTN2A1‐BTN3A1 ectodomain (PDB ID: 8DFX) [87]. (H) Crystal structure of the BTN3A1 ectodomain in complex with 103.2 scFv (PDB ID: 4F9P) [69]. (I) Crystal structure of the BTN3A1 ectodomain in complex with 20.1 scFv (PDB ID: 4F9L) [69]. (J) Molecular surface representation of the BTN3A1 ectodomain highlighting the binding footprints for 20.1 scFV (cyan) and 103.2 scFv (violet).

The discovery of BTN2A1 as an essential partner for BTN3A1 in pAg sensing prompted testing and validation of it as a direct ligand for the Vγ9Vδ2 TCR [85, 86]. Importantly, this interaction was independent of the Vδ chain and instead appeared to exclusively involve germline‐encoded regions of the Vγ9 chain since distinct Vγ9 TCR clonotypes bound BTN2A1, including Vδ2^neg^ TCRs [85, 86]. Mutagenesis implicated CDR2 and HV4 regions of the TCR Vγ9 chain, along with the CFG face of the BTN2A1 IgV domain, as critical components of the interface. In addition to predicting the BTN2A1 ectodomain structure as a V‐shaped, disulphide‐linked dimer, molecular modeling approaches predicted a TCR/BTN2A1 binding mode compatible with TCR recognition of BTN2A1 in trans [85] (Figure 5A). This mode is highly analogous to that proposed between Vγ4^+^ TCRs and BTNL3 [90], which was also dependent on germline‐encoded regions of the Vγ chain. Hence, the Vγ4/BTNL3 and Vγ9/BTN2A1 interactions exemplify a canonical non‐clonotypic Vγ/BTN binding mode, enabling surveillance of target cells for BTN presence or status. The subsequent crystallographic analysis of a Vγ9 TCR/BTN2A1 complex [87] confirmed the expected BTN2A1 V‐shaped dimer and confirmed an in trans TCR/BTN binding mode consistent with molecular modeling approaches (Figure 5A), albeit involving molecular contacts distinct from those predicted. Therefore, the molecular features of the TCR/BTN2A1 interaction explain the requirement for the Vγ9 chain in pAg sensing, and given the dimeric status of BTN2A1, leave unresolved the possibility that this binding mode facilitates clustering of the TCR. However, the binding mode does not involve other regions of the Vγ9Vδ2 TCR strongly implicated in pAg sensing. These include the CDR1/3 regions of Vγ9 and CDRs 1/2/3 regions of Vδ2, and their lack of involvement in the interaction with BTN2A1 strongly implicates additional ligands on the target cell as being critical players in the sensing process [85]. Finally, and potentially linking with a requirement for additional factors, surface plasmon resonance assays indicated the Vγ9Vδ2 TCR/BTN2A1 affinity is ~40‐50μM [85, 86], not dissimilar to weak αβ TCR/pMHC interactions, which typically fall within the 1–50 μM range. Interestingly, engagement of BTN2A1 (in the absence of BTN3A1) on target cells appeared to be sufficient to trigger basal activatory signaling in Vγ9Vδ2 T cells, but not full activation [85].

#### 2.4.2 TCR recognition of BTN3A IgV

The idea that the Vγ9Vδ2 TCR might interact directly with the ectodomain of BTN3A has been the subject of considerable debate. Such an interaction was hypothesized after the functional importance of BTN3A in both pAg‐mediated and immunostimulatory antibody‐mediated activation was highlighted [68]. However, the mechanisms through which pAg exert their stimulatory effects differ notably from those of the 20.1 mAb. Specifically, while the 20.1 mAb stimulated Vγ9Vδ2 activation by ligating the (highly similar) IgV ectodomains of either isoform of BTN3A (A1, A2, or A3), pAg‐mediated activation was critically dependent upon BTN3A1, particularly its intracellular regions, including the B30.2 domain, hypothesized as an important candidate site for pAg detection [68].

Despite this, a subsequent study employing x‐ray crystallographic and SPR analyses reported that the BTN3A1‐IgV domain could specifically bind pAg and present them at the cell surface, enabling enhanced interaction with the Vγ9Vδ2 TCR [73]. This finding was highly surprising, not least because the presentation of small molecules by IgV‐like domains is unprecedented in the whole of immunology. However, a later seminal study refuted these conclusions, explaining that the electron density attributed by Vavassori et al. to pAg bound to the BTN3A1‐IgV domain was instead likely due to an additive used in crystallisation [46]. Instead, and as outlined below, pAg was found to bind specifically to the BTN3A1 B30.2 domain (Figure 4D). Moreover, using soluble ectodomains, some of which were highly multimerised, Sandstrom et al. failed to detect γδ TCR/BTN3A interactions, and concluded that if such interactions occur, their Kd must be weaker than 500 μM. Subsequent studies have confirmed the core findings reported by Sandstrom et al., noting a lack of interaction of pAg with the BTN3A ectodomain [77], and corroborating direct pAg binding to BTN3A1‐B30.2 [74, 75, 76, 77].

Despite a failure to detect obvious TCR/BTN3A ectodomain interaction, the possibility remains that the Vγ9Vδ2 TCR may be able to interact with the BTN3A IgV domain via an extremely low‐affinity interaction. Although this remains unresolved, evidence from two recent studies supports this scenario. Willcox et al. used mutagenesis to identify an antigenic hotspot on the surface of the BTN3A IgV that was critical for pAg‐induced Vγ9Vδ2 activation [89] (Figure 5B, left panel). While the interaction of this patch with a third‐party protein cannot be excluded, the simplest hypothesis is that this hotspot is bound by the Vγ9Vδ2 TCR alongside TCR interaction with BTN2A1 as part of a composite ligand [89]. Secondly, Fulford et al. employed both Vγ9Vδ2 TCR tetramers and BTN2A1‐BTN3A1 heteromers, and mutagenesis to propose simultaneous TCR binding to BTN2A1 and BTN3A1 [87], highlighting residues on BTN3A1 that were proposed to contribute to interaction with the TCR (Figure 5B, right panel). Additional data establishing direct TCR/BTN3A IgV binding will be required to confirm such models.

#### 2.4.3 pAg‐Mediated BTN2A1/BTN3A1 B30.2 Interaction

The unequivocal demonstration of pAg binding to the BTN3A1 B30.2 domain [46], combined with the discovery of BTN2A1 as both an essential partner in pAg sensing and direct Vγ9Vδ2 TCR ligand [85, 86], prompted researchers to clarify the role of the intracellular domains of both proteins in the process. The Scotet group provided key insights into the role of the BTN3A1 intracellular region using chimeric BTN3A molecules, demonstrating that the juxtamembrane (JTM) domain plays a crucial role in the antigenic activation of human Vγ9Vδ2 T cells (Figure 5C, left panel) [91]. Expanding on this, Wang et al. proposed that BTN3A1 intracellular coiled‐coil dimers serve as rod‐like helical molecular spacers, positioning the B30.2 domains in a manner that is critical for sensing prenyl pyrophosphates by human Vγ9Vδ2 T cells (Figure 5C, right panel) [71]. Cano et al. used Forster resonance energy transfer (FRET) and proximity ligation assays to show that Zoledronate exposure increased the proximity of BTN2A1 and BTN3A1, including at the plasma membrane [80]. This FRET‐detected effect was dependent upon the BTN3A1‐B30.2 domain. Moreover, three independent studies established that pAg sensing by Vγ9Vδ2 T cells relied on the B30.2 domains of both BTN3A1 and, surprisingly, BTN2A1 [72, 80, 86]. However, whether such observations reflected direct or indirect interactions remained unclear. Building on these findings, the Weimer group employed ITC and nuclear magnetic resonance (NMR) spectroscopy to demonstrate a pAg‐dependent association between the intracellular domains of BTN2A1 and BTN3A1, with BRET experiments indicating that this binding event led to increased proximity at the cell surface [72]. Finally, they highlighted specific residues at the BTN2A1 juxtamembrane/B30.2 intersection as playing a key role in the association with pAg‐bound BTN3A1‐B30.2 (Figure 5D). These results strongly suggested that direct, antigen‐induced co‐association of BTN2A1 and BTN3A1‐B30.2 domains may be the critical pAg‐proximal event in the sensing process.

A seminal study by Yuan et al. subsequently outlined the structural basis of this pAg‐induced BTN3A1‐BTN2A1 B30.2 domain association [88]. X‐ray crystallographic analyses demonstrated that pAg binding to BTN3A1 formed a composite interface that enabled direct interaction with BTN2A1 (Figure 5E). Notably, structurally diverse pAgs can bridge this heteromeric interface, establishing contacts with both proteins. Thus, rather than BTN3A1 being viewed as the sole pAg sensor, this study underlined that pAg sensing is truly a cooperative process, requiring both BTN3A1 and BTN2A1, establishing the latter as a crucial component of the pAg sensor. Thus, the authors describe pAg as functioning like ‘molecular glue’ promoting heteromeric association of the intracellular domains of BTN3A1 and BTN2A1, a feature preserved for the BTN2A1/BTN3A molecules in Alpaca, the first non‐primate species demonstrated to retain a pAg‐reactive Vγ9Vδ2 T cell subset [41]. They speculated that the evolution of this antigen‐mediated, two‐protein interaction provides a highly sensitive mechanism by which the vertebrate immune system can detect subtle internal threats [88]. However, it remains less clear how this intracellular interaction triggers extracellular changes that culminate in productive Vγ9Vδ2 TCR/ligand interaction and triggering. One possibility, discussed in more detail in subsequent sections, is that the pAg‐induced heteromerisation event triggers conformational alterations that are transmitted extracellularly, disrupting BTN3A‐BTN2A1 IgV‐IgV interactions and initiating the formation of an activatory ligand.

#### 2.4.4 BTN2A1‐BTN3A1 Ectodomain Interaction

Studies identifying BTN2A1 as a critical additional cellular factor required for pAg sensing alongside BTN3A also sought to address whether this functional synergy was reflected by co‐localisation of the two proteins at the cell surface. Rigau et al. confirmed the proximity of BTN2A1 and BTN3A, both in the presence and absence of pAg, to within the 10 nm (i.e., 100 Å) resolution limit of FRET detection [86]. Karunakaran et al. established the co‐localisation of BTN2A1 and BTN3A ectodomains at the cell surface to within 16 Å, using a membrane‐impermeable chemical cross‐linker and immunoprecipitation approach, and found this too occurred in the presence and absence of pAg [85]. Based on this result, the authors hypothesized a direct interaction between the BTN2A1 and BTN3A1 ectodomains and confirmed weak IgV‐IgV‐mediated interactions using NMR [85].

Recent molecular studies have shed more light on the mode of BTN2A1‐BTN3A IgV‐IgV interaction and on the potential significance for Vγ9Vδ2 activation. NMR chemical shift analyses identified residues on the CFG face of both BTN3A and BTN2A1 as being involved (Figure 5F), with NMR‐informed models indicating a lateral, in cis binding mode [89] (Figure 5G, left). Importantly, while this lateral BTN2A‐BTN3A IgV‐IgV binding mode was consistent with chemical crosslinking studies, both modeling and NMR experiments indicated it was inconsistent with TCR interactions with BTN2A1, as the CFG face of BTN2A1 was involved in both interactions [89]. This indicated that BTN3A IgV and the TCR Vγ9 region represent ‘either/or’ ligands for the BTN2A1 IgV domain [89]. These findings were corroborated by Fulford et al., who, in addition to confirming the BTN2A1/BTN3A IgV‐IgV interaction was of a low affinity (~600 μM), generated a low‐resolution disulphide‐engineered BTN2A1‐BTN3A dimer to confirm an in cis binding domain [87] (Figure 5G, right). They also used mutagenesis and comparison with a Vγ9Vδ2 TCR/BTN2A1 crystal structure to similarly conclude the incompatibility of the two interactions [87].

Currently, the physiological importance and role of in cis BTN2A1‐BTN3A IgV‐IgV interactions are unclear. Given the incompatibility of such in cis interactions with the BTN2A1/Vγ9Vδ2 TCR interactions that are so critical to pAg sensing [87, 89], it is tempting to suggest that such in cis BTN2A1‐BTN3A heteromers may represent a TCR‐non‐reactive ‘ground state’ that, following pAg binding to intracellular B30.2 domains, is conformationally altered into an active conformation. Nevertheless, alternative possibilities exist, such as the lateral interactions helping to maintain the two proteins in close proximity, even in the absence of pAg [89]. In addition, the extremely weak nature of such in cis IgV‐IgV interactions, and their redundancy for effective pAg sensing in some experimental systems [89], leave their full physiological significance unclear, although they have been incorporated into some mechanistic models of pAg‐mediated Vγ9Vδ2 T cell activation.

#### 2.4.5 BTN‐Specific Immunostimulatory Antibody Interactions

The generation of BTN3A‐IgV‐specific antibodies with agonistic (20.1) or inhibitory (103.2) effects on Vγ9Vδ2 T cell activation, initially identified in functional assays of anti‐BTN3 antibodies [68, 92], represented an important development in the field, with the potential to shed light on underlying mechanisms of pAg sensing, and conceivably to impact therapeutically. Consequently, any robust mechanistic models of pAg sensing must accommodate the numerous observations regarding these reagents. The 20.1 mAb exhibited highly potent activatory effects, driving polyclonal Vγ9Vδ2 activation in a TCR‐dependent manner that, although independent of the mevalonate pathway, in certain respects appeared to mimic foreign pAg, by sensitizing target cells for recognition in some way. Its effects included triggering of Vγ9Vδ2 T cell Ca^2+^ release, CD69 upregulation, and both cytokine production (e.g., IFNγ, TNFα) and degranulation in Vδ2^+^ T cells, with no stimulatory effects observed on αβ T cells or Vδ2^neg^ γδ T cells [68]. In contrast, the 103.2 mAb inhibited Vγ9Vδ2 stimulation by pAg/NBP by binding to BTN3A on target cells, without exerting inhibitory effects on αβ T cell activation [68].

Numerous studies have attempted to address the molecular basis of 20.1 stimulatory and 103.2 inhibitory effects, and while uncertainty persists about core mechanisms, several important observations are noteworthy. A comprehensive molecular study addressed the binding mode of 103.2 and 20.1 (Figure 5HI–J) on BTN3A IgV, and also the stoichiometry, binding kinetics/affinity of interaction, and impact of valency on functional effects [69]. Notably, 103.2 inhibitory effects were abolished in the single‐chain variable fragment (scFv) format, indicating a steric hindrance mechanism of inhibition for the full‐length antibody, suggestive of masking important protein interactions with BTN3A [69]. In contrast, the activatory effects of 20.1 were evident in both Fab and scFv formats, albeit somewhat reduced. In addition, crystallographic comparisons of 20.1‐bound and unbound BTN3A IgV domain revealed no effect on the IgV domain conformation itself, but did indicate subtle rotational alterations in BTN3A V‐shaped dimer configuration upon 20.1 binding. These findings therefore do not completely exclude conformational change as a mechanism for 20.1 mAb‐induced activation, nor the alternative possibility that full‐length 20.1 mAb may exert some of its effects by clustering different BTN3A dimers. However, the findings clearly indicate that the binding of 20.1, even in scFv form, to the BTN3A IgV domain fundamentally enhances the latter's antigenicity.

An important clue to the 20.1‐mediated activatory mechanism is provided by the observation that despite potent stimulatory effects of the 20.1 mAb on primary Vγ9Vδ2 T cells, 20.1 exerts Vγ9Vδ2 TCR clonotype‐specific effects on activation, with some Vγ9Vδ2 TCRs incompatible with 20.1 stimulation [93]. Moreover, whereas pAg sensing is dependent on both BTN3A and BTN2A1, the 20.1 mAb can effectively sensitize rodent cells expressing BTN3A but lacking BTN2A1 for recognition by Vγ9Vδ2 T cells [84]. In addition, 20.1 can inhibit pAg‐dependent recognition [93]. These findings underscore fundamental differences in the modes of activation induced by 20.1 and pAg. Collectively, they suggest that 20.1 may, for permissive Vγ9Vδ2 clonotypes, augment an inherent potential for TCR engagement of BTN3A [93]. Depending on the experimental system and TCR clonotype involved, this may negate the requirement for coordinate TCR/BTN2A1 engagement. From this perspective, incompatibility of 20.1 mAb with some Vγ9Vδ2 clonotypes could either reflect steric occlusion by the antibody of the binding mode of certain specific TCR clonotypes on BTN3A IgV, or conceivably could reflect natural differences in the strength of TCR/BTN3A IgV interaction, with 20.1 stimulation requiring Vγ9Vδ2 TCRs of a certain threshold affinity.

## 3 Molecular Mechanism

### 3.1 Historical Perspective—Evolution of pAg‐Sensing Models

Pioneering work from multiple groups has established critical pillars of understanding of pAg sensing since the initial identification of alkyl phosphate antigens that recapitulated in vitro stimulation of Vγ9Vδ2 T cells by extracts from pathogens associated with in vivo expansions of this γδ T cell subset in human patients [3]. Crucially, these pillars have been critical in supporting our current mechanistic investigations (see Section 2.1). Nevertheless, understanding of the mechanisms underpinning pAg sensing has evolved considerably since its discovery. Arguably, the critical shift in thinking has been the transition from a cell surface to an intracellular role for the pAg itself. Early investigations favored direct recognition of pAg at the target cell surface by the Vγ9Vδ2 TCR, mediated by their presentation by an unidentified human‐specific antigen presentation molecule. As recently as 2013, BTN3A1 was proposed as the elusive cell surface molecule hypothesized to perform such a role [73]. However, as discussed previously, a now overwhelming body of evidence [46, 74, 75, 76, 77] indicates that instead of acting as direct antigens for TCR recognition analogous to MHC‐restricted peptides, pAg function intracellularly, as ‘indirect’ antigens, by binding to the intracellular B30.2 domain of BTN3A1. This proposes a different set of challenges for the field to understand, including chiefly how exposure of the cytosolic region of BTN3A1 to intracellular pAg is communicated to the extracellular face of the membrane, and what the nature of the ligand complex recognized by the TCR comprises. Below we summarize and explain the key current concepts that address one or both of these questions. Nevertheless, the dual necessity for both intracellular and extracellular BTN3A domains for efficient pAg sensing suggests the existence of a completely novel mechanism that facilitates sensitive and extremely fast ‘inside‐out’ signaling of pAg exposure by target cells to Vγ9Vδ2 T cells via their TCR.

### 3.2 Composite Ligand

The concept that the Vγ9Vδ2 TCR might recognize a ‘composite’ ligand, consisting of elements comprised of more than one molecule, first emerged after studies that identified BTN2A1 as a direct target for Vγ9Vδ2 TCR binding [85, 86]. Since Vγ9Vδ2 TCR‐mediated pAg sensing is thought to involve CDRs from both the Vγ9 and Vδ2 chains [50], the demonstration by both studies that TCR/BTN2A1 interaction was dependent only on germline‐encoded regions of the Vγ9 TCR chain indicated that other TCR/ligand interactions must be involved. Subsequent work has shed light on the nature of the composite ligand.

One possibility was that an additional, non‐butyrophilin ligand was present on the target cell surface, whose localization was perhaps influenced by pAg interaction with the BTN3A1‐B30.2 domain. However, attempts to identify such a molecule have been unsuccessful, disfavoring this hypothesis [89]. A second possibility was that the composite ligand comprised elements from both BTN2A1 and BTN3A, aligning with the heteromer model outlined below. The discovery of the ‘in cis’ lateral binding mode between BTN2A1 and BTN3A1‐IgV domains [89] suggested that such a complex of the two proteins could in principle form such a composite ligand. However, a combination of NMR, modeling, and mutagenesis experiments, later confirmed by separate crystallographic and mutagenesis experiments, indicated that TCR recognition of BTN2A1 involves a region of the BTN2A1‐IgV domain that overlaps substantially with the patch involved in ‘in cis’ interaction with BTN3A IgV [89]. Therefore, the TCR/BTN2A1 interaction established as central to pAg sensing is incompatible with ‘in cis’ BTN2A1‐BTN3A1 interaction [89]. This finding suggested that the lateral ‘in cis’ BTN2A1‐BTN3A1 IgV‐IgV interaction, which is extremely weak, is unlikely to synergize with TCR/BTN2A1 as a component of an activatory composite ligand [89]. One superficially attractive hypothesis is that the ‘in cis’ BTN2A1‐BTN3A1 IgV‐IgV interaction could represent an important but non‐activatory ‘ground state’ that is subsequently altered following pAg‐induced intracellular BTN2A1‐BTN3A1 association. Furthermore, a mutation that eliminated ‘in cis’ BTN2A1‐BTN3A1 IgV‐IgV interaction, albeit in a cellular system in which BTN2A and BTN3A were overexpressed, had no effect on pAg induced activation [89], indicating this ‘ground state’ is most likely not essential to the pAg sensing process. Overall, the significance of ‘in cis’ BTN2A1‐BTN3A1 IgV‐IgV interaction is unclear. An alternative proposal is that it could play a role in ensuring proximity of BTN2A1 and BTN3A dimers at the cell surface in the absence of pAg, thereby facilitating rapid responses upon pAg exposure by limiting the requirement for extensive lateral diffusion [89]. This could be more significant at lower, physiological expression levels of BTN2A1 and BTN3A, but the notion that such weak lateral interactions affect their cell surface proximity is supported by the observation of decreased BRET signals between BTN2A1 and BTN3A molecules upon deletion of the ectodomain regions [80].

In the absence of compelling novel non‐BTN ligand candidates, and given that TCR‐mediated recognition of the ‘in cis’ BTN2A1‐BTN3A1 IgV‐IgV interaction is not central to pAg sensing [40], the most plausible alternative suggestion was that the composite ligand is comprised of simultaneous but spatially distinct TCR interactions with both BTN2A1‐IgV and BTN3A‐IgV [40]. As outlined below, this possibility is supported by the identification of an ‘antigenic hotspot’ on BTN3A1‐IgV that critically affects pAg sensing but plays no role in the ‘in cis’ interaction with BTN2A1, and which was postulated as a potential direct interaction surface for the TCR [40]. Nevertheless, given previous studies on TCR/BTN3A1 interaction [46], this interaction would likely have to be very weak but might be sufficient in the context of TCR/BTN2A1 interaction to induce TCR triggering. Furthermore, additional work by Fulford et al. [87] lends credence to this possibility, providing some evidence for weak TCR/BTN3A interaction and for the existence of two distinct ligand interaction surfaces on the TCR; one involving germline‐encoded regions of the Vγ9 chain, for interaction with BTN2A1, and the other, including at least some contributions from Vδ2 amino acids, mediating interaction with BTN3A IgV.

In summary, given the involvement of diverse CDR regions in Vγ9Vδ2 TCR‐mediated pAg sensing [50] and the purely germline‐encoded binding mode of TCR/BTN2A1 interaction [85, 86], there are compelling reasons to believe that pAg sensing is underpinned by TCR recognition of a composite ligand, most likely a combination of epitopes on BTN2A1 and, separately, BTN3A1 [87, 89]. Moreover, although the composite ligand model has largely been described in the context of extracellular TCR‐mediated recognition events, it is envisaged that it can be reconciled with studies demonstrating pAg‐induced association of BTN3A1 and BTN2A1 B30.2 domains, as outlined below.

### 3.3 Molecular Glue

The concept of molecular glues first emerged from work on Cyclosporin A (CspA), a naturally occurring small molecule originally identified from soil microbes, subsequently developed as an immunosuppressive drug [94]. Studies investigating CspA's mechanism of action revealed that it bound to Cyclophilin, enabling the latter to interact with and inhibit the protein phosphatase calcineurin, thereby dampening downstream inflammatory pathways. The relevance of the concept has since expanded to encompass a range of biological systems, with the term ‘molecular glue’ now generally applied to small molecules that exert potent biological effects by binding to a target protein and critically altering its interactome. Potential downstream consequences include stabilizing or destabilizing target proteins, altering their localization, or triggering signaling by inducing proximity to interactors [94].

Recent seminal work from the Zhang group, which defined the structural basis of pAg‐induced BTN3A1‐B30.2/BTN2A1‐B30.2 association [88], suggested the molecular glue concept is likely applicable to pAg sensitization of target cells. This built on previous pivotal findings from Hsiao et al., who first demonstrated pAg‐dependent association of the BTN2A1 and BTN3A1 B30.2 domains [72]. Ground‐breaking crystallographic studies of Yuan et al. clarified that by binding to a positively charged pocket on BTN3A1, pAg can bridge a heteromeric interface between the B30.2 domains of BTN3A1 and BTN2A1, forming direct contacts with residues from both proteins [88]. Combined with solution‐based binding experiments that established the exquisite dependence of BTN2A1‐B30.2/BTN3A1‐B30.2 interaction on the presence of pAg [72, 88], these data justify classification of pAg as molecular glues. Moreover, they highlight exploitation of the molecular glue phenomenon by the vertebrate immune system as a means to detect and initiate responses to microbial infection, in contrast to microbes that leverage this phenomenon to promote specific host protein interactions in order to drive persistence and immune evasion [94]. In doing so, these recent findings [88] arguably redefine the pAg‐sensor as truly a two‐protein system, establishing that BTN3A1 and BTN2A1 are directly involved in what is likely the most pAg‐proximal event in the sensing process.

Despite these findings, it is currently unclear how the intracellular ‘molecular glue’ action of pAg to induce B30.2‐mediated BTN3A1/BTN2A1 interaction is propagated to the extracellular surface to flag up relevant target cells as antigenically exposed. Importantly, the structural data from the Zhang group appear to exclude large pAg‐induced conformational changes in the B30.2 domain of either BTN3A1 or BTN2A1 [88]. In the absence of pAg, it is possible that ‘in cis’, IgV‐IgV‐mediated ectodomain interactions between BTN2A1 and BTN3A [89] ensure proximity of the two proteins, but represent a TCR‐non‐reactive ground state. Yuan et al. proposed that following pAg association with BTN2A1/BTN3A1, rigidification of the BTN3A1 B30.2 domain led to the propagation of structural perturbations to the extracellular face of the complex, which may be sufficient to disrupt the TCR‐non‐reactive ‘in cis’ BTN2A1‐BTN3A IgV‐IgV ‘ground state’ [88]. While intriguing, this suggestion was based on inconclusive evidence from molecular dynamics, NMR, and atomic force microscopy approaches. An important additional caveat is that the ‘in cis’ BTN2A1‐BTN3A IgV‐IgV interactions are inherently very weak [87, 89] and may spontaneously form and dissociate. Therefore, while the molecular glue paradigm nicely describes the highly pAg‐specific heterodimerization of BTN2A1 and BTN3A1 B30.2 domains, a broader understanding of how this is linked to extracellular changes is currently lacking.

### 3.4 Heteromer Recognition

Since the dual importance of BTN3A and BTN2A1 in pAg sensing was recognized in 2020 [85, 86], the possibility that the TCR might bind to both targets has been postulated. While TCR interaction with BTN2A1 was quickly established and its functional importance confirmed, validating direct TCR interaction with BTN3A has been challenging. Before the discovery of BTN2A1 as a Vγ9‐dependent TCR ligand, TCR/BTN3A interaction was proposed to occur in a pAg‐dependent manner [73], although this suggestion was later refuted by Sandstrom et al. [46], who took extensive steps to explore the possibility of direct TCR/BTN3A interactions but concluded this was unlikely to occur with an affinity greater than 500 μM. Nevertheless, the possibility remains that direct TCR/BTN3A interactions occur that are extremely weak, but in the context of the existing TCR/BTN2A1 interaction, nevertheless functionally critical.

Recent published data either corroborate or actively support TCR co‐binding a heteromer of BTN2A1 and BTN3A1. Notably, Willcox et al. [89] and Karunakaran et al. [81] highlighted the importance of the IgV domain of BTN3A molecules in pAg sensing, with the former using mutagenesis to establish a critical ‘antigenic hotspot’ on its surface [89]. Moreover, Willcox et al. proposed lateral ‘in cis’ BTN2A1/BTN3A1 IgV‐IgV interactions, but their modeling and NMR studies indicated that these were incompatible with TCR/BTN2A1 binding. Collectively, these results were consistent with the TCR coordinating separate, weak, but synergistic interactions with BTN2A1‐IgV and BTN3A1‐IgV, although these results did not exclude alternative models.

Fulford et al. built on these and earlier studies, using crystallographic approaches to confirm a TCR/BTN2A1 binding mode broadly in keeping with previous predictions [87], and to confirm (via a cysteine‐cysteine mutant form) an ‘in cis’ BTN2A1‐IgV/BTN3A1‐IgV interaction similar to that proposed by Willcox et al. [87]. They also corroborated earlier conclusions [89] that the BTN2A1‐IgV/BTN3A1‐IgV interaction was incompatible with the TCR/BTN2A1 interaction [87]. Importantly, they carried out a series of multimer binding experiments, consisting of either TCR‐tetramer binding to target cells expressing BTN2A1 and BTN3A1, and heteromer multimers of BTN2A1 linked by leucine zippers to BTN3A1, in conjunction with mutagenesis of both the TCR and BTN2A1‐IgV/BTN3A‐IgV [87]. These results led them to propose two binding surfaces exist on the TCR; one, involving germline encoded regions of Vγ9 dedicated to engagement of BTN2A1, and another, predominantly focused on Vδ2, involved in binding BTN3A [87]. Mutagenesis of BTN3A defined a patch likely involved in engagement of the latter binding site. Moreover, one Vδ2 mutant of the TCR, K53A, exhibited enhanced reactivity to BTN3A, suggesting a potential ‘gate‐keeper’ role in this interaction. Finally, precomplexing BTN3A‐IgV with 20.1 mAb was found to increase TCR tetramer staining, consistent with a direct TCR/BTN3A‐IgV interaction [87].

Collectively, these results provide powerful evidence for TCR/BTN3A interaction, albeit requiring additional confirmatory data.

### 3.5 Conformational Change and Dimerisation

The role of conformational changes in BTN2A1/BTN3A1 following pAg binding as a mechanism of ‘inside‐out’ signaling to the Vγ9Vδ2 TCR is currently unclear. Nevertheless, in principle, an antigen‐induced conformational change is an attractive mechanism that might plausibly translate the intracellular signal provided by pAg exposure to the extracellular, TCR‐binding face of the BTN2A1/BTN3A1 complex.

In this context, numerous studies have assessed evidence for conformational alterations and differences in dimerisation status within the ectodomains or cytosolic domains of BTN2A1 and BTN3A1 that might relate to the activatory mechanism. Crystallographic analyses have demonstrated the potential for distinct conformations of the BTN3A1 extracellular domains. Specifically, a V‐shaped dimer formed through IgC‐IgC contacts has been observed in the crystal lattice of BTN3A1, A2, and A3 ectodomain dimers, and its dominant presence in solution in the steady state is supported by FRET and cryo‐EM 2D class averages and likely reflects forms at the cell surface [69]. In contrast, no evidence was obtained that a ‘head‐to‐tail’ dimer of the BTN3A ectodomain observable in the crystal lattice was present in solution in the steady state [69]. Nevertheless, a caveat is that such studies were based on analysis of the BTN3A ectodomain in isolation. Subsequent studies have suggested that the ‘head‐to‐tail’ dimer conformation may indeed form at the cell surface in the context of full‐length BTN3A molecules, potentially representing an inactive state that allows the molecule to lie flat against the cell membrane and remain inaccessible to the γδ‐TCR [70].

In addition, the intracellular domain of BTN3A and BTN2A1 has been observed to form homodimers in crystal structures. Structures of the intracellular B30.2 domains of BTN3A1 [46, 70] and BTN2A1 [88] revealed two distinct, mutually exclusive dimer configurations: one exhibiting two‐fold symmetry with an interface distant from the pAg‐binding pocket and the other asymmetric, involving a pAg‐proximal domain of one B30.2 molecule interfacing with a pAg‐distal domain of another [70]. Zhang and colleagues proposed the functional importance of the asymmetric BTN3A1‐B30.2 dimer by demonstrating that bulky analogues of HMBPP, despite their higher affinity for the BTN3A1 B30.2 domain, exhibited significantly weaker Vγ9Vδ2 activation activity, suggesting that these analogues interfere with dimer formation through steric hindrance [95]. Based on their findings, they proposed that the functional intracellular asymmetric dimer corresponds to the extracellular ‘head‐to‐tail’ dimer involved in γδ T cell activation.

Several caveats are worth highlighting in relation to such observations. Firstly, caution is required when interpreting interaction surfaces observed in crystallographic approaches, which necessarily involve protein–protein contact sites to form the crystal lattice. Employing tangential approaches to independently validate or invalidate findings is essential. Such solution‐based validation approaches appear to disfavor the head‐to‐tail dimer of the BTN3A ectodomain [69]. Secondly, the fact that BTN3A dimers may often comprise highly functional heterodimers incorporating BTN3A1 paired with BTN3A2 [81, 82], which lacks a B30.2 domain, emphasizes that dimers of the BTN3A1‐B30.2 domain (whether symmetric or asymmetric) are unlikely to be an essential feature of the pAg sensing mechanism. Finally, Yang et al.'s suggestion that the decreased potency of bulky pAg analogues is due to disruption of asymmetric BTN3A1 B30.2 dimer formation [95] does not take into account their later findings regarding pAg‐induced interaction with BTN2A1 [88], which is likely disrupted by such larger pAgs.

The evidence for pAg‐induced conformational alterations in BTN3A1/BTN2A1 is also intriguing. Several studies focusing on recombinant BTN3A1 endodomain in isolation suggest that pAg binding induces some conformational alterations. The first indication of such effects was the dissolution of BTN3A1‐B30.2 crystals following soaking with pAg, likely due to structural alterations that disrupted crystal packing [46]. Subsequent NMR studies by Hsiao et al. reported chemical shift perturbations (CSPs) in the BTN3A1 intracellular domain upon pAg addition, indicating significant changes in the chemical environment of specific residues [74]. Further investigations confirmed that such pAg‐induced CSPs occurred not only in residues localized near the pAg‐binding pocket but also extended to regions distal from it, suggesting global conformational shifts within the B30.2 domain upon pAg binding [70, 74, 77]. In addition to the observed CSPs in the B30.2 domain, the Wiemer group identified significant pAg‐induced changes in the juxtamembrane (JM) region, distinct from the pAg binding pocket [74]. The Scotet group further demonstrated by mutagenesis that alterations in this region can markedly enhance or diminish T cell reactivity, underscoring the functional significance of the JM region in γδ T cell activation [91].

Despite these results, the recently described structure of BTN2A1‐B30.2 in complex with pAg‐bound BTN3A1‐B30.2 indicates minimal, if any, detectable conformational alterations between unbound forms of BTN2A1‐B30.2 and BTN3A1‐B30.2 and their pAg‐associated form [88]. Resolution of these apparently discordant observations might be provided by molecular dynamics approaches, which indicate a rigidification of the BTN3A1‐B30.2 domain following pAg binding, which could underlie both the disruption of apo BTN3A1‐B30.2 crystals by pAg soaking [46] and pAg‐induced NMR CSPs [70, 74, 77]. Importantly, the lack of significant conformational alteration in either BTN2A1 B30.2 or BTN3A1 B30.2 upon binding would appear to strongly disfavor a simplistic conformational change model as the primary mechanism of pAg‐induced inside‐out signaling. Instead, it suggests that a rigid connection between the B30.2 domains and the external face of each butyrophilin, facilitated by the coiled‐coil domains, might be key in transmitting intracellular pAg‐induced BTN2A1/BTN3A1 association to the extracellular face of the plasma membrane.

Evidence for pAg‐ or activation‐induced changes to the BTN3A1/BTN2A1 ectodomain conformations is currently minimal, necessitating further work in this area. Crystal structures of the immunostimulatory 20.1 mAb in complex with BTN3A proteins have demonstrated no conformational alterations within the BTN3A‐IgV domains [69]. In addition, as this antibody has immunostimulatory effects even in scFv format [69], it seems unlikely that clustering of separate BTN3A dimers is solely driving its effects. Of note, in structures of BTN3A with 20.1 scFv, a change in the rotational angle of BTN3A monomers within the V‐shaped BTN3A dimer led to a ~20 Å shift in BTN3A IgV position relative to the apo BTN3A structure [69]. However, this may simply reflect a degree of conformational plasticity of the isolated BTN3A ectodomain in solution, and the differential propensity of specific rotamers for forming crystal contacts in the presence/absence of 20.1 scFV. Especially given the evidence for Vγ9Vδ2 TCR contact with BTN3A IgV (see above), it seems most likely that explanations other than conformational/rotational alterations in the BTN3A‐IgV domains, such as the formation of a BTN2A1‐independent BTN3A‐Fv composite ligand for the Vγ9Vδ2 TCR, underlie 20.1 immunostimulatory capacity, and might substitute for pAg‐induced alignment of BTN2A1 and BTN3A ectodomains as part of a composite ligand. A more likely focus for pAg‐induced conformational alterations is the disruption of lateral, in cis IgV‐IgV interactions of the type initially detected and modeled by the Willcox group. Since such interactions are incompatible with TCR/BTN2A1 interaction [89], they might conceivably represent a non‐activatory ‘ground state’ of the BTN2A1‐BTN3A complex. Therefore, pAg‐induced disruption of such interactions might feasibly promote TCR/BTN2A1 and conceivably TCR/BTN3A interactions. Although additional studies are required to address how these events might be coupled, the Zhang group proposed that pAg‐mediated BTN2A1 association drives BTN3A1 intracellular fluctuations outward toward the extracellular regions of BTN2A1/BTN3A1, and suggested this enables BTN3A1‐IgV to disengage from the BTN2A1‐IgV ectodomain, thereby initiating TCR‐mediated activation of γδ T cells [88]. This suggestion was based on multiple approaches including structure‐based molecular dynamics simulations, ^19^F‐NMR, chimeric receptor engineering, and atomic force single cell microscopy [88].

Collectively, these data indicate that a conceptually simple pAg‐induced conformational change in the BTN3A1‐B30.2 domain may not be the predominant mechanism that drives the formation of an activatory BTN2A1‐BTN3A complex capable of initiating Vγ9Vδ2 TCR triggering.

### 3.6 Topological Considerations

Although previous data highlight the importance of both the ectodomains and endodomains of both proteins in pAg sensing, the topological requirements for activatory BTN2A1‐BTN3A complex formation have remained unclear. Crucially, in humans, the btn complex comprises a single btn2a1 gene and three btn3a genes, encoding BTN3A1, A2, and A3 [53]. This contrasts with species such as the Alpaca, which possess a single btn3a gene [41]. Previous studies have shown that, although BTN3A2 and BTN3A3 lack a functional B30.2 domain, their co‐expression with BTN3A1 is important for efficient pAg sensing [76, 81, 82]. Both molecules are capable of heterodimerizing with BTN3A1, and it has been hypothesized that their chaperone function enhances trafficking efficiency to the cell surface relative to BTN3A1 and is an important factor underlying their ability to boost BTN3A1‐mediated pAg sensing [81, 82].

A recent study expanded on these findings, employing an extensive mutagenesis approach to shed new light on the topological requirements for BTN2A1/BTN3A/pAg mediated activation [81]. Intriguingly, domain deletion experiments revealed that in the context of BTN3A1‐BTN3A2 or BTN3A1‐BTN3A3 heterodimers, the BTN3A1‐IgV was expendable, whereas the IgV domain of either BTN3A2 or BTN3A3 was essential. Additionally, specific amino acid differences within a charged section of the juxtamembrane region of BTN3A molecules favored BTN3A1 heterodimeric pairing with BTN3A2/A3, and contributed to the inefficiency of BTN3A1 homodimer‐mediated pAg sensing, rationalized by poor intermolecular pairing in the coiled‐coil regions [81]. In contrast, the juxtamembrane region of Alpaca BTN3A is thought to facilitate efficient cell surface trafficking, homodimer formation, and pAg‐dependent complexation with BTN2A1, potentially representing a primordial pAg‐sensing BTN3A molecule [41]. Collectively these results establish that there is a clear ‘division of labour’ within the human BTN3A dimer, with BTN3A1 critically providing intracellular B30.2 pAg‐sensing capability, and its heterodimeric partner chain (either BTN3A2 or BTN3A3) providing a non‐redundant IgV domain for TCR‐mediated recognition. They suggest topological constraints for a Vγ9Vδ2 TCR/ligand complex, with potential for cross‐over of BTN3A monomer chains permitting the TCR to engage a central B30.2‐mediated complex of BTN2A1 and BTN3A1 [81, 89].

### 3.7 T Cell Second Ligand Models

The potential of BTN3A to mediate additional Vγ9Vδ2 TCR‐independent interactions with T cell‐expressed ligands was suggested by Vyborova et al. [96], based on binding experiments involving BTN3A‐coated beads. Although this potentially aligns with previous work highlighting BTN3A interaction with T cells [92, 97], staining was limited to Jurkat cell lines, leaving the identity of the putative ligand and its importance for pAg sensing unclear. Nevertheless, these data were used to propose a two‐receptor, three‐ligand model of pAg sensing, involving parallel Vγ9Vδ2 TCR interaction with BTN2A1, potentially a separate as‐yet‐undefined second target‐cell‐expressed ligand, and finally, BTN3A‐mediated interaction with a second receptor on the T cell surface [96]. While this model is highly speculative, it may hold relevance, particularly in light of findings by Payne et al. [98], who reported that BTN3A1 engages with CD45 on the T cell surface, which was proposed to prevent CD45 segregation from the vicinity of the TCR, thereby dampening TCR signaling. Although these findings are intriguing, their significance for either the core pAg sensing mechanism or, more broadly, for BTN‐mediated modulation of T cell responses in general, is currently unclear.

## 4 Prospects for an Integrated Mechanistic Model

A central challenge in the field is to incorporate those critical observations from the diverse studies that have addressed the same fundamental pAg sensing system in order to construct an integrated mechanistic model of the process. Here below, we outline our own attempt to do this.

### 4.1 Toward a Unified Theory of pAg Sensing

**FIGURE 6:**
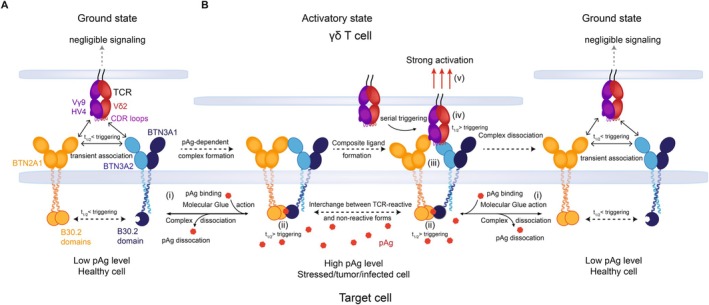
The MaGiCaL Heteromer model of pAg sensing. (A) In the ground state, low affinity TCR/BTN2A1 or TCR/BTN3A1 interactions can in principle take place, but each is insufficiently strong and stable for substantial TCR triggering; concurrently, any lateral *in cis* BTN2A1‐BTN3A1‐IgV‐IgV associations are weak and transient; any B30.2 domain‐mediated BTN2A1/BTN3A1 complexes are extremely short‐lived, and ultimately permit lateral diffusion of each BTN away from each other. (B) The ‘molecular glue’ action of intracellular pAg on BTN3A1 and BTN2A1 B30.2 domains (i) results in BTN3A/BTN2A1 complexes of defined stability, colocalising their ectodomains (ii) upon which dissociation of (low affinity) BTN2A1‐BTN3A1 IgV‐IgV ectodomain interactions (if present) opens up a composite ligand (iii) permissive for TCR interaction (iv), with binding affinity/kinetics sufficient to effect TCR triggering (v). The schema shown does not exclude the possibility of a pAg‐bound BTN2A1/BTN3A complex featuring IgV‐IgV interaction incompatible with TCR interaction, which upon dissociation of IgV‐IgV interaction could convert to a form allowing TCR/composite ligand interaction. The *t*_1/2_ of pAg association with the BTN2A1/BTN3A1 B30.2 complex determines the lifetime of the composite ligand and its ability to carry out serial triggering of the Vγ9Vδ2 TCR‐CD3 complexes on the T cell surface, accounting for differential potency of HMBPP (stable BTN3A1 and BTN2A1 association, high potency) and IPP (weaker BTN3A1 and BTN2A1 association, lower potency). The strength of TCR signaling is likely a product of both the stability of the pAg‐BTN2A1/BTN3A1 complex (and therefore on the inherent binding capability of the pAg itself), and the intracellular pAg concentration. Dissociation of pAg leads to disassembly of stable BTN2A1/BTN3A1 complexes, and a return to the ‘ground state’ (A).

In recent years, the field has arguably been converging on such an integrated pAg sensing mechanism. In outlining key steps in this mechanism here (below and Figure 6), we have combined elements of many of the models discussed above, while deliberately omitting elements of some models we feel have either been superseded or may not be central to the core sensing process. Any robust mechanism should ultimately be able to incorporate the action of immunomodulatory antibodies directed at relevant BTN family members, and that is discussed in Section 2.3.1.

- Intracellular binding of pAg to the BTN3A1‐B30.2 domain triggers coassociation with the BTN2A1‐B30.2 domain (Molecular Glue).
- Co‐association of BTN2A1 and BTN3A dimers is propagated through the membrane via relatively rigid coiled‐coil domains of the two proteins.
- The ectodomains of BTN2A1 and partner‐BTN3A are colocalized in a manner that, under physiological conditions, permits coordinated but spatially distinct interaction with a single TCR.
- The Vγ9Vδ2 TCR binds simultaneously to a composite ligand comprising residues on the CFG face of BTN2A1 (via Vγ9) and to BTN3A‐IgV (via regions including those on Vδ2). Whereas the binding surface on BTN2A1 is recognized by Vγ9 independent of clonotype, clonotypic differences may influence the exact TCR footprint on BTN3A‐IgV (Composite ligand).
- The combination of epitopes on BTN2A1‐IgV and BTN3A‐IgV enables more stable TCR binding, permitting full TCR triggering to take place.

The above scheme primarily combines elements of the Composite Ligand [81, 85, 89] (Section 3.2) and Molecular Glue (Section 3.3) [72, 88] concepts, but accommodates the increasing evidence for direct TCR interaction with both BTN2A1 and BTN3A interactions (outlined in Section 3.4 on Heteromer Recognition [87]) and for the importance of topological constraints on the activatory complex [81] (Section 3.6). From this perspective, a possible name for the model is the Molecular Glue‐Composite Ligand Heteromer model, or (MaGiCaL Heteromer model). Although substantial structural information is required to validate the model, as envisaged, pAg‐induced conformational change is not invoked as a central element in the mechanism, nor is the involvement of additional ligands on the T cell surface, nor is TCR dimerization, aligning with recent results indicating Vγ9Vδ2 TCRs are monomeric when isolated as TCR/CD3 complexes [48].

### 4.2 Support for the MaGiCaL Heteromer Model From Recent Cryo‐EM Studies

A recent cryo‐EM study, likely to be bolstered by additional studies, has provided critical support for the MaGiCaL heteromer model. Zhang et al. determined full‐length structures of BTN2A1 in complex with a BTN3A1/BTN3A2/pAg heteromer, both in the absence and presence of a soluble Vγ9Vδ2 TCR [99].

Importantly, the study validates the composite ligand concept, highlighting that the TCR simultaneously interacts with separate elements on the BTN2A1‐IgV and BTN3A2‐IgV domain surfaces. Specifically, the interaction with BTN2A1‐IgV exclusively involves germline‐encoded components of the Vγ9 chain, while the interaction with BTN3A2‐IgV encompasses contributions from both TCR chains, but is dominated by elements of the Vδ2 chain. This finding aligns with predictions derived from mutational dissection of the topology of the activatory complex, indicating that the BTN3A2‐IgV domain directly interacts with the TCR [81], whereas the BTN3A1‐IgV domain is redundant [81]. In contrast, the BTN3A1‐B30.2 domain is critical, bridging a central pAg‐mediated interaction with the BTN2A1 B30.2 domain, as highlighted by previous studies which emphasized the molecular glue action of pAg [88]. Therefore, the TCR‐liganded structure of pAg‐associated BTN2A1 and BTN3A1 appears to confirm many key tenets of the MaGiCaL Heteromer model outlined above. Importantly, as well as providing an initial glimpse of the TCR regions involved in composite ligand formation, it highlights some conformational alterations in regions of the Vδ2 TCR chain involved in binding BTN3A2‐IgV. Although the current dataset does not establish to what extent such changes may be clonotype‐specific, such changes might be expected for such a low affinity interaction, as a requirement for the TCR to bind in particular conformational forms could impose a substantial energetic penalty upon binding, akin to αβ TCR/pMHC interactions [100]. In summary, such changes in the TCR may reflect an unusual interaction evolved for low affinity binding, rather than conformational alterations likely to initiate TCR triggering.

The structure of the pAg‐associated BTN2A1/BTN3A complex in the absence of TCR [99] is surprising in two respects. Firstly, the complex fails to highlight any pAg‐induced conformational changes transmitted to the ectodomains that would favor the assembly of an activatory composite ligand. It is important to note, however, that no apo structure of BTN3A dimers were determined, which would limit the interpretation of these results. Conversely, and surprisingly, the pAg‐associated but TCR‐unliganded structure indicates the BTN2A1 and BTN3A2 ectodomains form an ‘in cis’ IgV‐IgV interaction that precludes TCR binding. This superficially counter‐intuitive result seems to exclude the possibility that pAg‐mediated association of the BTN2A1/BTN3A1 B30.2 domains necessarily results in an inside‐out conformational change that dissociates the BTN2A1 and BTN3A IgV ectodomains in readiness for TCR binding, as previously suggested [99]. How then can this TCR‐unliganded structure be reconciled with the compelling evidence that pAg‐triggered association of BTN2A1 and BTN3A1 drives the formation of an activatory complex, and with the cryo‐EM evidence that this involves the spatially separated IgV domains of BTN2A1 and BTN3A forming distinct, but coordinated interactions with the TCR? A plausible interpretation might take account the fact that this static TCR‐non‐reactive in cis structure was determined at 100 K and could simply reflect one low energy state of the BTN2A1 and BTN3A ectodomains. Especially given the extremely weak affinity of the IgV‐IgV interaction, the experimental conditions may fail to adequately capture the range of possible ectodomain conformers present under physiological conditions, which likely include both the in cis dimer observed in this structure and configurations where BTN2A1‐IgV and BTN3A‐IgV are dissociated in a manner compatible with cooperative interaction with the TCR. This scenario appears more likely than an alternative suggestion that TCR engagement somehow triggers dissociation of the ‘in cis’ BTN2A1/BTN3A IgV‐IgV dimer [99]. While this work leaves the basis of pAg‐induced inside‐out signaling unclear, it appears to disfavor conformational change as a primary mechanism. Instead, it focuses attention on pAg‐induced co‐localization of BTN2A1 and BTN3A ectodomains as the principal driver of activatory composite ligand formation.

### 4.3 Reflections on A Unified Theory

The emergence of a unified mechanism of Vγ9Vδ2 TCR‐mediated pAg sensing, as outlined above, allows for comparison and interpretation of its quintessential features in the broader context of other biological systems.

In the model of pAg sensing outlined above, the positive output, that is, TCR triggering, is only achieved upon close co‐localisation of BTN3A and BTN2A ectodomains, allowing simultaneous TCR interaction with each protein. In the absence of pAg, the model predicts that the lack of stable heterodimerisation between the two dimers results in TCR interaction with either BTN2A1 or BTN3A, resulting in a largely negative output (i.e., lack of or only basal TCR signaling). From this perspective, the mechanism could be viewed as analogous to a specialized form of logical AND gate, a concept that has been applied to the design of CAR‐T cells that integrate CD3zeta‐based signals and costimulatory signals from distinct engineered receptors to increase selectivity for particular targets [101]. In the case of pAg sensing, the system primarily reports not merely on the presence of the two target components (BTN2A1 and BTN3A) but rather on their molecular proximity. Moreover, as opposed to behaving like a binary on/off switch, importantly, the sensing apparatus is capable of responding differently to quantitatively distinct triggering pAg signals, since foreign pAg such as HMBPP are detected at far lower concentrations than their endogenous counterpart IPP. While much remains to be learned concerning the molecular details of the mechanism, the underlying biophysics of the component interactions are likely to be critical to the sensing process. These include pAg affinity for BTN3A1/BTN2A1, which likely affects the *t*_1/2_ of the heteromeric activatory complex. In this regard, the substantially higher affinity of HMBPP than IPP for the BTN3A1‐B30.2 domain would predict that HMBPP gives rise to far longer‐lived heteromeric activatory complexes. In addition, the affinity of the component TCR/BTN2A1 and TCR/BTN3A interactions, and the affinity/kinetics of the Vγ9Vδ2 TCR interaction with the activatory ligand complex, and indeed the pAg concentration, are likely to be key determinants of the overall outcome.

A second analogy that could be made is with TCR/pMHC interaction. This might initially be surprising, given the drastic structural differences between the two ligand systems. However, energetically, the two systems are arguably somewhat aligned, given both αβ TCR/pMHC recognition and Vγ9Vδ2 pAg sensing rely on augmenting TCR interaction with a core target (MHC or BTN2A1, respectively) by the addition of a second component (antigenic peptide, or co‐localized BTN3A IgV, respectively). In each case, the second component is likely crucial in tipping the TCR/ligand interaction over a critical biophysical threshold required for TCR triggering.

Previously, an analogy has been drawn between Vγ9Vδ2‐mediated pAg sensing and pattern recognition receptor recognition (PRR) of pathogen‐associated molecular patterns (PAMPs), which underlie innate immune sensing and responses to diverse bacterial and viral components [18]. In this comparison, the B30.2 domains of BTN2A1 and BTN3A1 are analogous to the PRR, whereas bacterial pAg such as HMBPP qualify as microbe‐specific PAMPs. Such conventional PRR/PAMP systems typically involve multiple steps between PAMP detection and the initiation of the downstream protective response. In contrast, the pAg sensing mechanism outlined above is an extremely parsimonious PRR/PAMP detection system since the same molecular pair (BTN2A1/BTN3A) directly couples PAMP/pAg detection (via the Molecular Glue mechanism) with the assembly of an activatory Composite Ligand that, by triggering Vγ9Vδ2 TCR, is directly responsible for initiating the downstream protective response.

Finally, the unified mechanism outlined above provides a useful perspective from which to understand the mechanism of action of antibodies directed at BTN3A that have potent immunomodulatory effects on Vγ9Vδ2 T cell activation. Firstly, the inhibitory action of the BTN3A‐specific 103.2 mAb, which is much less potent as a Fab/scFv [69], can be rationalized as sterically blocking Vγ9Vδ2 TCR interaction with BTN3A IgV. Perhaps more intriguing are the potent immunostimulatory effects of the BTN3A‐specific 20.1 mAb, which is retained substantially in both Fab and scFv formats [69], indicating the core mechanism of action is not related to clustering BTN3A molecules. Based on the observations that the 20.1 mAb does not change the conformation of BTN3A‐IgV (as evidenced by structural studies) [69] and can exert immunostimulatory effects in some systems in the absence of BTN2A1 [84], a likely mechanism is that the mode of action of 20.1 involves augmenting TCR affinity for BTN3A IgV. While additional studies are required in this area, this may operate by 20.1 introducing a novel binding surface for the Vγ9Vδ2 TCR, increasing TCR/BTN3A‐IgV interaction sufficiently to achieve a stability/affinity that surpasses a threshold required for TCR triggering. Hence, 20.1 may synergize with BTN3A IgV to create an alternative, BTN2A1‐independent activating composite ligand for the Vγ9Vδ2 TCR. In the context of the unified mechanism outlined above, this proposed mode of action of 20.1 could account for the clonotype‐dependent stimulatory effects this antibody can elicit [93], since whereas the antibody may augment TCR/BTN3A IgV interaction for some Vγ9Vδ2 clonotypes, other clonotypes may engage the BTN3A‐IgV surface in such a way as to sterically clash with the 20.1/BTN3A interaction.

## 5 Future Directions

### 5.1 Unanswered Questions

Our current state of understanding of Vγ9Vδ2 TCR‐mediated pAg sensing presents several unresolved questions that warrant further investigation.

An obvious but important one relates to the architecture of the complex formed when the Vγ9Vδ2 TCR engages its activatory composite ligand, comprising elements of BTN2A1 and BTN3A, which remains the subject of much ongoing interest. In relation to this, validating direct TCR interaction with BTN3A‐IgV and understanding the role of the various CDR regions in ligand recognition would represent significant advances in the field. Upcoming cryo‐EM studies (including [99]) should contribute usefully to resolving these issues.

A second, interrelated question concerns the degree of plasticity accommodated in the TCR/ligand complex in the context of different TCR clonotypes present within the semi‐invariant Vγ9Vδ2 TCR repertoire and the functional implications of such different footprints. Understanding to what extent Vγ9Vδ2 clonotype‐specific cellular reactivity to infected or cancerous cells is determined by differences in TCR clonotype affinity for the activatory ligand complex could yield important insights with potential therapeutic implications.

In addition, the molecular mechanism by which immunomodulatory antibodies affect Vγ9Vδ2 TCR‐mediated pAg sensing is currently unclear and warrants further study. Resolving the nature of the TCR/activatory ligand complex will undoubtedly help formulate modes of action of relevant antibodies. Insights from such advances may help explain how antibodies exert clonotype‐dependent effects and may clarify whether some BTN‐specific immunostimulatory antibodies operate by forming alternative activatory composite ligands.

However, arguably the question most central to pAg sensing is how the intracellular association of pAg with the BTN3A and BTN2A B30.2 domains triggers the assembly of the extracellular composite ligand bound by the TCR. Cryo‐electron microscopy approaches [99] are uniquely suited to shed light on this question, since they could define full‐length structures of the critical players, namely BTN2A1 and BTN3A, ideally in the presence and absence of pAg and TCR. While such studies should shed light on ‘inside‐out’ BTN2A1/BTN3A‐mediated signaling in response to pAg exposure, it is essential to acknowledge caveats regarding extrapolating interpretations from structures obtained at 100 K to recognition events at the cell surface. A particularly pressing issue relating to this is the degree to which pAg exposure triggers inside‐out conformational changes in the ectodomains of BTN2A1/BTN3A, or alternatively whether the system primarily signals antigenic exposure by B30.2‐mediated/pAg‐induced lateral co‐localization of BTN2A1/BTN3A ectodomains. In relation to the latter possibility, it will be crucial to achieve a high‐resolution understanding of the cell surface distribution of BTN2A1 and BTN3A molecules, as well as how this changes over time and in response to pAg exposure. Such insights will be important in unequivocally establishing the pAg sensing mechanism.

Finally, the roles of non‐butyrophilin proteins in pAg sensing are of ongoing interest, with both periplakin, a cytoskeletal adaptor protein, and RhoB, a small GTPase, implicated in the process. Periplakin has been shown to interact with the cytosolic domain of BTN3A1 via a short membrane‐proximal di‐leucine motif, a feature of BTN3A1 required for the restoration of the pAg‐dependent response in BTN3A1 knockdown cell lines [76]. Furthermore, the activation of RhoB in target cells has been implicated in boosting pAg‐mediated sensitisation and has been suggested to affect the spatial redistribution of BTN3A1, facilitating the compartmentalization of BTN3A1 to membrane‐proximal areas, thereby enhancing its interaction with pAgs and promoting T cell activation [54, 102]. Further investigation of how each of these proteins contributes to the dynamic regulation of BTN3A1 during pAg sensing is warranted.

### 5.2 Relevance for Other Subsets

The relevance of the Vγ9Vδ2 TCR/BTN2A1/BTN3A‐mediated pAg sensing paradigm to that of other systems is currently unclear. However, tissue‐restricted expression of pairs of BTN family molecules also includes BTNL1 and BTNL6 in the mouse intestine [103], and the orthologous BTNL3 and BTNL8 in the human intestine [103], as well as Skint‐1 [65] and Skint‐2 [104] in mice skin. Moreover, Btnl1 (in mice) and BTNL3 (in human) have been shown to interact with germline‐encoded regions of Vγ7 and Vγ4 TCR chains, respectively, in a manner very analogous to the TCR Vγ9/BTN2A1 interaction [90]. The potential clinical significance of such interactions is emphasized by recent findings regarding human null alleles of BTNL3, which have been suggested to associate with increased susceptibility to certain forms of inflammatory bowel disease [105]. Consequently, the Vγ9Vδ2 pAg sensing mechanism, with regulated colocalization of distinct BTN dimers at its core, may prove to be a template to which these other systems align, thereby rationalizing the co‐expression of BTN family pairs in diverse biological settings. Of note, it is possible that BTN2A1 and BTN3A primarily exist in isolation in the steady state and in the pAg‐induced associated state under microbial/non‐microbial stress. However, in principle, one could envisage a system operating in reverse, with constitutive BTN/BTN interaction giving rise to homeostatic signaling that was disrupted under stress. Irrespective of the directionality of stress sensing, the presence of cytoplasmic B30.2 domains in BTNL3/8 and BTNL1/6 suggests that moieties akin to pAg may trigger activation of these systems. In contrast, Skint‐1 and Skint‐2 lack B30.2 domains, and while both are important for DETC selection and immunosurveillance in the skin [65, 104, 106], the extent of alignment with Vγ9Vδ2 stress sensing is unclear. In summary, much further research is required to define underlying mechanisms in these related but microenvironmentally highly distinct systems.

## Conflicts of Interest

B.E.W. consults for Ferring Ventures regarding γδ T cell immunotherapy development, including related to Vγ9Vδ2 T cells. The other authors have no conflicts of interest related to the topics discussed in this review.

## Data Availability

Data derived from public domain resources.
